# Diagnosis of Myalgic Encephalomyelitis/Chronic Fatigue Syndrome With Partial Least Squares Discriminant Analysis: Relevance of Blood Extracellular Vesicles

**DOI:** 10.3389/fmed.2022.842991

**Published:** 2022-04-01

**Authors:** Alba González-Cebrián, Eloy Almenar-Pérez, Jiabao Xu, Tong Yu, Wei E. Huang, Karen Giménez-Orenga, Sarah Hutchinson, Tiffany Lodge, Lubov Nathanson, Karl J. Morten, Alberto Ferrer, Elisa Oltra

**Affiliations:** ^1^Grupo de Ingeniería Estadística Multivariante, Departamento de Estadística e Investigación Operativa Aplicadas y Calidad, Universitat Politècnica de València, Valencia, Spain; ^2^Department of Pathology, School of Health Sciences, Universidad Católica de Valencia San Vicente Mártir, Valencia, Spain; ^3^Nuffield Department of Women's and Reproductive Health, The Women Centre, University of Oxford, Oxford, United Kingdom; ^4^Department of Engineering Science, University of Oxford, Oxford, United Kingdom; ^5^Escuela de Doctorado, Universidad Católica de Valencia San Vicente Mártir, Valencia, Spain; ^6^Kiran C. Patel College of Osteopathic Medicine, Nova Southeastern University, Fort Lauderdale, FL, United States; ^7^Institute for Neuro Immune Medicine, Nova Southeastern University, Fort Lauderdale, FL, United States; ^8^Centro de Investigación Traslacional San Alberto Magno, Universidad Católica de Valencia San Vicente Mártir, Valencia, Spain

**Keywords:** myalgic encephalomyelitis/chronic fatigue syndrome (ME/CFS), extracellular vesicles (EVs), partial least squares-differential analysis (PLS-DA), Raman spectroscopy, microRNAs, carotenoids, biomarker

## Abstract

Myalgic Encephalomyelitis/Chronic Fatigue Syndrome (ME/CFS), a chronic disease characterized by long-lasting persistent debilitating widespread fatigue and post-exertional malaise, remains diagnosed by clinical criteria. Our group and others have identified differentially expressed miRNA profiles in the blood of patients. However, their diagnostic power individually or in combinations seems limited. A Partial Least Squares-Discriminant Analysis (PLS-DA) model initially based on 817 variables: two demographic, 34 blood analytic, 136 PBMC miRNAs, 639 Extracellular Vesicle (EV) miRNAs, and six EV features, selected an optimal number of five components, and a subset of 32 regressors showing statistically significant discriminant power. The presence of four EV-features (size and *z*-values of EVs prepared with or without proteinase K treatment) among the 32 regressors, suggested that blood vesicles carry relevant disease information. To further explore the features of ME/CFS EVs, we subjected them to Raman micro-spectroscopic analysis, identifying carotenoid peaks as ME/CFS fingerprints, possibly due to erythrocyte deficiencies. Although PLS-DA analysis showed limited capacity of Raman fingerprints for diagnosis (AUC = 0.7067), Raman data served to refine the number of PBMC miRNAs from our previous model still ensuring a perfect classification of subjects (AUC=1). Further investigations to evaluate model performance in extended cohorts of patients, to identify the precise ME/CFS EV components detected by Raman and to reveal their functional significance in the disease are warranted.

## Introduction

Myalgic Encephalomyelitis/Chronic Fatigue Syndrome (ME/CFS) is a highly debilitating disease characterized by unexplained profound fatigue lasting over 6-months (ICD-10 code R53.82 or G93.3 if post-viral) (1), which is exacerbated by physical, mental, or emotional activity, a process known as post-exertional malaise (PEM); lack of restoring sleep, dysautonomia, and frequent additional comorbidities (2). Despite recent intense biomarker research, its diagnosis relies on clinical symptom assessment, after excluding potential underlying health problems that could relate to patient symptoms (3–5).

Historically, pathway biomarkers that have been interrogated in ME/CFS include cytokine profiles, immune cell subpopulations and metabolites, as reviewed by Maes et al. (6). The recognized value of microRNAs as liquid biopsy biomarkers of complex disease (7, 8) led to genome-wide screenings of miRNA profiles in ME/CFS blood fractions by ours and other research groups (9–12). These encouraging findings, however, have so far failed to provide a specific biomarker signature of the disease (6). Extracellular vesicles (EVs) released by most cell types in the organism can be collected from blood potentially reporting information of the entire organism physiology. This was the reason for our previous study to evaluate ME/CFS EVs and their miRNA contents. Although altered levels of overlapping markers were found for some miRNAs from PBMCs and EVs (12), no miRNA has been widely validated as a biomarker of ME/CFS, and all identified so far appear to have limited diagnostic value, individually or when combined.

Rudimentary statistical methods such as two sample tests (i.e., *t*-test or Wilcoxon-Mann Whitney test), followed by multiple comparison corrections [i.e., Bonferroni or False Discovery Rate (13)] for the analysis of “omic” data have several drawbacks. These include low statistical power, lack of interpretability of results, and the omission of complex relationships among variables which could, in principle, be addressed using other statistical approaches such as linear or generalized linear models. However, these methods suffer from other problems when dealing with “omic” data, such as large number of variables and low sample size, which produces overfitting, and the high correlation among variables, which produces multi-collinearity. Those limitations have motivated the development of numerous novel statistical techniques (14).

Prediction methods such as Partial Least Squares (PLS) (15) is one of these novel techniques especially suitable for the analysis of “omic” data due to its ability to deal with more variables than observations, and its good model interpretation capacity (16). Conceived as an alternative to classical regression, PLS, is a statistical multivariate technique that models the latent space of predictors and responses (X and Y subspaces, respectively) finding the subspace which maximizes the covariance between both latent subspaces. PLS-DA (Discriminant Analysis) is a variant of PLS for binary responses (17). The work we are presenting here used PLS-DA approaches to classify individuals in the healthy control (HC) or the case group, but also to determine which variables hold best discriminant power between these two classes of participants.

The study includes three PLS-DA models. The first was applied to over 800 variables obtained from 15 severe ME/CFS female cases and 15 matched HCs from the UK ME/CFS Biobank (UKMEB). Data included subject phenotyping with validated instruments, complete blood analytics, miRNA profiles from peripheral blood mononuclear cells (PBMCs) and from plasma-isolated extracellular vesicles (EVs), plus EV associated features, as previously described (9). The results showed that a combination of 32 variables, including several EV features, best discriminates severe ME/CFS cases from healthy subjects. The value of EV features for the assessment of ME/CFS was further supported by Raman spectroscopic data.

The second PLS-DA model focused on detecting discriminant regions of the Raman spectra. These results were compared with classification based on Raman spectra using three other binary classification techniques: an adaptation of linear discriminant analysis (LDA) (18) to deal with more variables than observations, random forest (RF) (19) and support vector machines (SVM) (20).

Finally, the relevant regions of the discriminatory spectra were included in a third PLS-DA model with the previously mentioned set of 32 variables. Using this approach, ME/CFS EV differences detected by Raman helped to further refine our previous ME/CFS PLS-DA model reducing the number of required miRNAs from PBMCs and further supporting the EV potential biomarker value for the diagnosis of ME/CFS.

To the best of our knowledge, this study is the first to provide a PLS-DA model for the accurate diagnosis of severe ME/CFS based on a discreet combination of variables. In addition, we used for the first time Raman fingerprints of EVs to enhance the ability to discriminate severely affected ME/CFS patients from healthy subjects.

## Materials and Methods

### Samples and Associated Clinical Data

Ethical approval of the study was granted by the Public Health Research Ethics Committee DGSP-CSISP, Valencia (Spain), study number UCV_201701 and by the UCL Biobank Ethical Review Committee-Royal Free London NHS Foundation Trust (B-ERC-RF), study number EC2017.01 before the samples were released by the UKMEB.

Data for the initial PLS-DA analysis corresponded to Nanostring datasets generated during a previous study of our group (12), available from the NCBI Gene Expression Omnibus (GEO) database (Accession Number GSE141770) and the (supplementary material) of the cited article. The samples for the Raman analysis consisted of EV aliquots from the cited study isolated from 0.5 ml of platelet poor plasma from 15 severely ill ME/CFS females and 15 age-population matched healthy females, obtained from dipotassium EDTA blood-collection tubes by UKMEB professionals.

As previously described, patient recruitment and clinical assessment for the UKMEB was mainly performed through the UK National Health Service (NHS) primary and secondary health care services (9). Compliance with the Canadian Consensus (4), CDC-1994 (“Fukuda”) (3) and Institute of Medicine (21) criteria were ensured for patient recruitment (22, 23). Clinical diagnosis was complemented with score differences in the SF-36 questionnaire (24) and the GHQ (General health Questionnaire) (25), the last also assessed by a Likert scale (9, 26).

Participants exclusion criteria were as follows: (i) take antiviral medication or drugs known to alter immune function in the preceding 3 months (ii) had any vaccinations in the preceding 3 months; (iii) had a history of acute and chronic infectious diseases such as hepatitis B and C, tuberculosis, HIV (but not herpes virus or other retrovirus infection); (iv) another chronic disease such as cancer, coronary heart disease, or uncontrolled diabetes; (v) a severe mood disorder; (vi) been pregnant or breastfeeding in the preceding 12 months; or (vii) were morbidly obese (BMI ≥ 40).

All methods were performed in accordance with relevant guidelines and regulations. All subjects signed an informed consent before samples could be included in the corresponding sample collection.

### Partial Least Squares-Discriminant Analysis (PLS-DA)

In this work we used three PLS-DA models: a first multi-block (27) PLS-DA model (Section PLS-DA Model to Classify ME/CFS Identifies EV Features as Potential Disease Biomarkers), a Raman-based PLS-DA model (Section ME/CFS Classification Model Based on Raman Spectral Fingerprints) and a second multi-block PLS-DA model (Section Refinement of the Initial PLS-DA Model With EV Raman Profiles). It is important to mention that different schemes of calibration and validation were used.

The multi-block PLS-DA models had two goals: to obtain an accurate classifier usable with new individuals and to interpret the set of discriminant features. Given the small sample size of the database, we followed a two-steps procedure. First, we used all observations (i.e., participants) to fit a PLS-DA model obtaining a set of statistically significant discriminant predictors. This way, most observations could be used to fit the PLS-DA model, reducing the uncertainty in the estimation of the parameters of the model, which is a critical aspect for the interpretation goal. Secondly, the dataset was split into calibration and validation subsets. The PLS-DA model was fitted using the relevant predictors of observations from the calibration subset and the model was then used to predict new observations from the validation set. Eight randomly selected individuals were included in the validation subset (four ME/CFS cases and four HCs). For preprocessing, a multi-block approach with block scaling and variable autoscaling was applied. Each block contained a different group of variables with similar features. Five blocks were established: (i) Demographic Variables, (ii) Analytic Variables, (iii) PBMCs' miRNA expression levels, (iv) EVs' miRNA expression levels, and (v) EVs' characteristics (9). The second multi-block PLS-DA model included an additional block with relevant Raman profile features.

For the Raman spectra PLS-DA model, the goal was to determine if an accurate diagnostic tool could be developed solely based on Raman spectra differences. It was crucial to compare all classifiers not only in terms of classification performance, but also in terms of model stability. For this reason, the chosen setup consisted of a three-fold cross-validation scheme. Each fold contained 1/3 of the data, i.e., each fold contained a set of 10 observations (five of each class). In each round, two-folds were used to fit the model and the other fold was used as an external validation set. This way, all observations were used to fit and validate the model, studying the stability on its performance. In this model, the preprocessing consisted of variable centering.

The performance of PLS-DA models was evaluated by the *R*^2^ coefficient (goodness of fit) and the *Q*^2^ coefficient (goodness of prediction). Permutation tests were used to assess the statistical significance of the model using the SIMCA software. A permutation test (28) consists in randomly permuting the values of the response, yielding a randomized data structure. Afterwards, a new PLS-DA model is fitted using the randomized response, obtaining its corresponding *R*^2^ and *Q*^2^ coefficients. The values for the *R*^2^ (and *Q*^2^) coefficients obtained in a series of different permutation testing yields the null distribution of the *R*^2^ (and *Q*^2^) coefficients under the assumption of no discrimination between both classes. Thus, this permutation framework also offers the possibility of calculating *p*-values associated with testing the hypothesis of model discrimination. Additionally, to evaluate the classification performance of the model, the Receiver Operating Characteristic (ROC) curve was obtained. For each ROC curve, the AUC (Area Under the Curve) was calculated (29).

Beyond its performance, one of the advantages of PLS-DA models is their interpretability. The PLS (*b*) coefficients coefficients represent the direct relationship between the original predictors' subspace (X) and the response categories (Y). The higher a *b* coefficient of a variable is (in absolute value), the more discriminant that predictor will be. The sign of the coefficient indicates the type of the relationship between the variable and the class to be predicted (negative or positive relationship). For the parameters and outcomes of the PLS-DA model, statistical significance was assessed by jackknife intervals at a 95% confidence level. These intervals are calculated in a cross-validation scheme implemented by the Aspen ProMV software used to obtain the PLS-DA model.

Once a PLS-DA model is fitted, it is quite common to follow an iterative depuration procedure variable-wise and observation-wise. On one hand, it is frequent to find that some predictors are not relevant. This can occur when the confidence interval of a *b* coefficient contains a zero value. In this case, it is possible to perform an initial variable selection, retaining only the relevant predictors to refit the PLS-DA model. For this variable selection the *b* coefficients and the Variable Importance for the Projection (*VIP*) coefficients, are used. *VIP* coefficients (30) represent the influence of each predictor, accounting its weight in each of the latent variables and the percentage of variability of the Y matrix explained by each latent variable. The threshold value of ≥1 for the *VIP* coefficients is a common threshold to identify variables which are potentially important in the model. Thus, predictors having a *VIP* with a confidence interval clearly under the 1 value and *b* coefficients not statistically significant were removed from the modeling.

In this work, the iterative depuration of predictors also helped to reduce uncertainty of the model estimates by decreasing the number of parameters of the model.

On the other hand, it is also common to perform an iterative model fitting until a PLS-DA model without outliers and relying only in relevant predictors, is obtained. Outliers were studied in terms of the Squared Prediction Error (SPE) and Hotelling's T^2^ (31) metrics.

Finally, to confirm and visualize the discriminant properties of the selected variables (i.e., those showing statistical significance in the PLS-DA) a two sample t- test was applied a posteriori to each potential biomarker included in the final multi-block PLS-DA model. These results can be found in the Supplementary Material.

### Isolation of EVs From Plasma

EVs studied corresponded to aliquots isolated from 0.5 ml aliquots of human plasma supernatants from blood collected in dipotassium EDTA tubes (Becton Dickinson, Franklin Lakes, NJ, USA) (undergoing a single freeze/thaw cycle), upon being centrifuged at 10,000 × g for 10 min, with Total Exosome Isolation Reagent (TEIR) (Invitrogen by Life Technologies, Cat. 4484450), following manufacturer's recommendations, as previously described (12). The isolated EVs were characterized following MISEV (Minimal information for studies of extracellular vesicles) recommendations (32), as described in Almenar-Pérez et al. (12).

### Raman Spectroscopy

After dilution of the isolated EVs to a concentration of 5 × 10^8^ EVs/ml in distilled water, 1.5 μL of the suspension was deposited on aluminum Raman slides and exposed to room temperature until the sample was completely dry. Spectra were acquired using an HR Evolution confocal Raman microscope (Horiba Jobin-Yvon, UK, Ltd.) equipped with a 532 nm laser. Laser power was 4.5 mW and a filter of 25%. The acquisition time per spectrum was 3 s at a resolution of 4 μm.

For the analysis of the Raman spectra, all spectra were preprocessed by cosmic ray correction, polyline baseline correction, and area normalization using the entire spectral region, using LabSpec 6 (Horiba Scientific, France). Data analysis, statistics and visualization were carried out using in-house scripts in *R*. Quantification of important biomolecules was performed by integrating the corresponding Raman bands. The quantification results were represented as box plots and sample means of the patients were compared with HCs by using Welch's two sample *t*-test for unequal variance.

Four classification models were trained with a three-fold cross validation setup to classify a spectrum as either severe ME/CFS or HC using an adaptation of linear discriminant analysis (LDA) (18) to deal with more variables than observations, random forest (RF) (19), a support vector machine (SVM) (20), and PLS-DA. For the LDA, RF, and SVM models, the classifier learning app in MATLAB was used, enabling the optimization of model hyperparameters. The AUC was calculated for each model, enabling the comparison of their classification performance.

### Pathway and Gene Enrichment Analysis

Analysis of predicted and validated miRNA-mRNA interactions was performed with the freely available software MiRTargetLink 2.0 (https://www.ccb.uni-saarland.de/mirtargetlink2) (33). Gene ontology (GO) enrichment analysis was performed using the miEAA tool incorporated into MiRTargetLink 2.0, targets were retrieved, sorted by adjusted *p*-value, and presented in table format. Selected networks of mRNAs targeted by at least two miRNAs were drawn using Adobe Illustrator software.

## Results

As described in a previous study (12), study participants were women with an average age of 46.8 (age range 38–53) for the disease cohort and 45.2 (age range 18–52) years for the matched HC group. Median ages were 48 years and 47 for the ME/CFS and HC group, respectively. Average time from disease onset was 17.5 (range 1.5–30.9) years, with a median value of 18.4 years. Health survey SF-36 and General Health Questionnaire (GHQ) scores, including Likert scale for the GHQ, scores clearly separated ME/CFS and HC groups (*p* < 0.05). Score details can be consulted in the referred work by Almenar-Pérez et al.

### PLS-DA Model to Classify ME/CFS Identifies EV Features as Potential Disease Biomarkers

Given the small sample size of the cohort, this first PLS-DA modeling step focused on finding the most statistically significant biomarkers for identifying the severe ME/CFS subjects. All observations (i.e., participants) were used to fit the model in an attempt to reduce as much as possible the uncertainty in the estimation of the model parameters.

#### ME/CFS Modeling With PLS-DA

ME/CFS PLSA-DA was performed on a collection of data obtained from 30 participants (15 severe ME/CFS females and 15 healthy subjects matched by sex and age (±5 y) of the UKMEB, as previously reported by our group (12) [Nanostring datasets available from the NCBI Gene Expression Omnibus (GEO) database, Accession Number GSE141770]. The complete set of data included 34 blood analyte variables, 775 miRNAs expressed above threshold levels (136 in PBMCs and 639 in EVs), EV concentration, size and *z*-potential of vesicles prepared with and without proteinase K treatments for a total of six EV-associated measures, together with two demographic variables. The 15 variables obtained from the SF-36 questionnaire (24) and the GHQ questionnaire (25), the last also assessed by a Likert scale (26) were not included since a diagnostic based solely on objective measurements was pursued.

The initial model was fitted with three latent variables (obtained by cross-validation) with a cumulative value of 96% for the *R*^2^ coefficient (goodness of fit) and 68% for the *Q*^2^ coefficient (goodness of prediction). After obtaining the PLS-DA model, we checked for potential outliers, removing subjects with an SPE (i.e., Euclidean distance to the model) overpassing the control limit (an example of outlier can be seen in Supplementary Figure 1).

The initial PLS-DA model presented a large number of predictors having a *VIP* with a confidence interval clearly below 1 and non- statistically significant b coefficients (Supplementary Figure 2). Thus, after performing an iterative variable selection, as described in Section Materials and Methods, the final model with the most discriminant variables was obtained.

**Figure 1:**
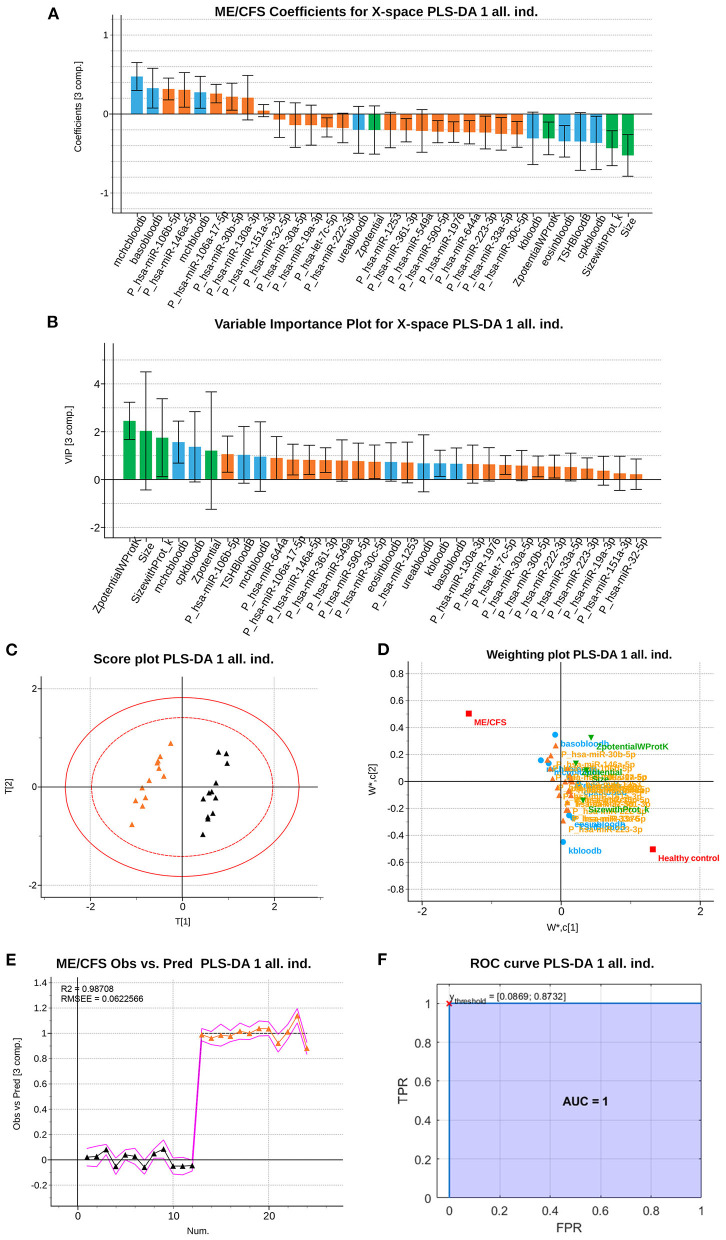
Partial Least Squares (PLS)-Discriminant Analysis (DA) multiblock model based on 32 variables measured from 12 ME/CFS patients and 12 HCs. **(A)** ME/CFS class jackknife *b* coefficients for the X subspace. The color code corresponds to the block each variable belongs to, being those analytical variables (blue), PBMCs' miRNAs (orange) and EVs' characteristics (green). Jackknife confidence Intervals were calculated at a 95% confidence level. **(B)** *VIP* coefficients with jackknife confidence intervals at 95% of confidence for the X subspace using the calibration dataset. Data set legends can be consulted on Supplementary Table 1. The color code is the same as in the rest of the figures. **(C)** Score plot, of the 1st and 2nd components (horizontal and vertical, respectively). **(D)** Weighting plot, of the 1st and 2nd components (horizontal and vertical, respectively). The color code for each variable block is the same as in the rest of the figures. **(E)** Observed vs. Prediction results for participants shows the class prediction with 95% confidence intervals (magenta lines) using three components. The color code is orange for ME/CFS patients, and black markers for Healthy Controls (HCs). RMSEE stands for Root Mean Square Error of Estimation. **(F)** ROC curve for the classification of the observations with the dataset. The red cross locates the optimal performance point (maximum specificity and sensitivity) using the classification threshold between 0.0869 and 0.8732.

This depurated PLS-DA model with 32 variables (Figures 1A,B) had similar cumulative *R*^2^ and *Q*^2^ values (98.71 and 96.31%, respectively), and the optimal number of components based on cross-validation was three (as the initial model). This model was based on a set of *N*=24 observations, having 12 of each class.

The permutation test illustrated in Supplementary Figure 3 shows that the *R*^2^ and *Q*^2^ values of the obtained PLS-DA model (points belonging to the 100% correlation between original y and permuted y) are greater than any of those belonging to the permuted datasets. Thus, the statistical significance of the 98.71 and 96.31% values for the *R*^2^ and *Q*^2^, respectively, is accepted, rejecting the hypothesis of having obtained these values by chance (with *p* < 0.05).

Furthermore, the stability and reliability of the final PLS-DA model in terms of its prediction performance can be visualized both in the scores scatterplot (Figure 1C) and in the observed vs. prediction plot (Figure 1E).

The score scatterplot (Figure 1C), showing a clear separation between groups, is directly related with the weighting plot (Figure 1D), which shows the correlation structure between the original and the latent variables. Thus, the probability of being a severe ME/CFS individual (orange triangle in the score scatterplot) is positively correlated with the variables at the same side (left) of the weighting plot, which are the same variables with a positive *b* coefficient for the ME/CFS class. This means that those variables tend to have greater values in ME/CFS than in HCs. Analogously, the set of variables placed at the opposite semi plane (right part) of the weighting plot (with negative *b* coefficients for the ME/CFS class), are negatively correlated to the probability of belonging to the ME/CFS class. This means that these variables tend to have lower values in ME/CFS than in HCs. Finally, variables near to the origin (0,0) point are those with coefficients not statistically different from zero (i.e., no statistical differences in both groups of participants).

Finally, the observed vs. prediction results for the participants showed a class prediction with 95% confidence intervals (magenta lines) using just three components, allowing all 12 patient observations to be correctly classified in the ME/CFS group and all 12 observations from healthy subjects in the HC group (Figure 1E). The ROC curve of the model shows a perfect classification of the samples (Figure 1F), since the AUC for both classes reach a value of 1. This means that the model has a perfect sensitivity and specificity (both equal to 1), i.e., it detects all patients and differentiates all controls as healthy individuals.

#### Classification Performance of the PLS-DA Model With Calibration and Validation Set

The second modeling approach focused on evaluating the potential of our PLS-DA model as a tool to correctly assign new observations into ME/CFS and HC groups. For this second PLS-DA model, the database was partitioned in a training and validation subsets, as explained in the Section Materials and Methods.

The trained model with three components (the same number as the previous model with all the observations) reaches cumulative values of 99.32% for the goodness of fitting coefficient (*R*^2^) and 88.52% for the goodness of prediction coefficient (*Q*^2^).

**Figure 2:**
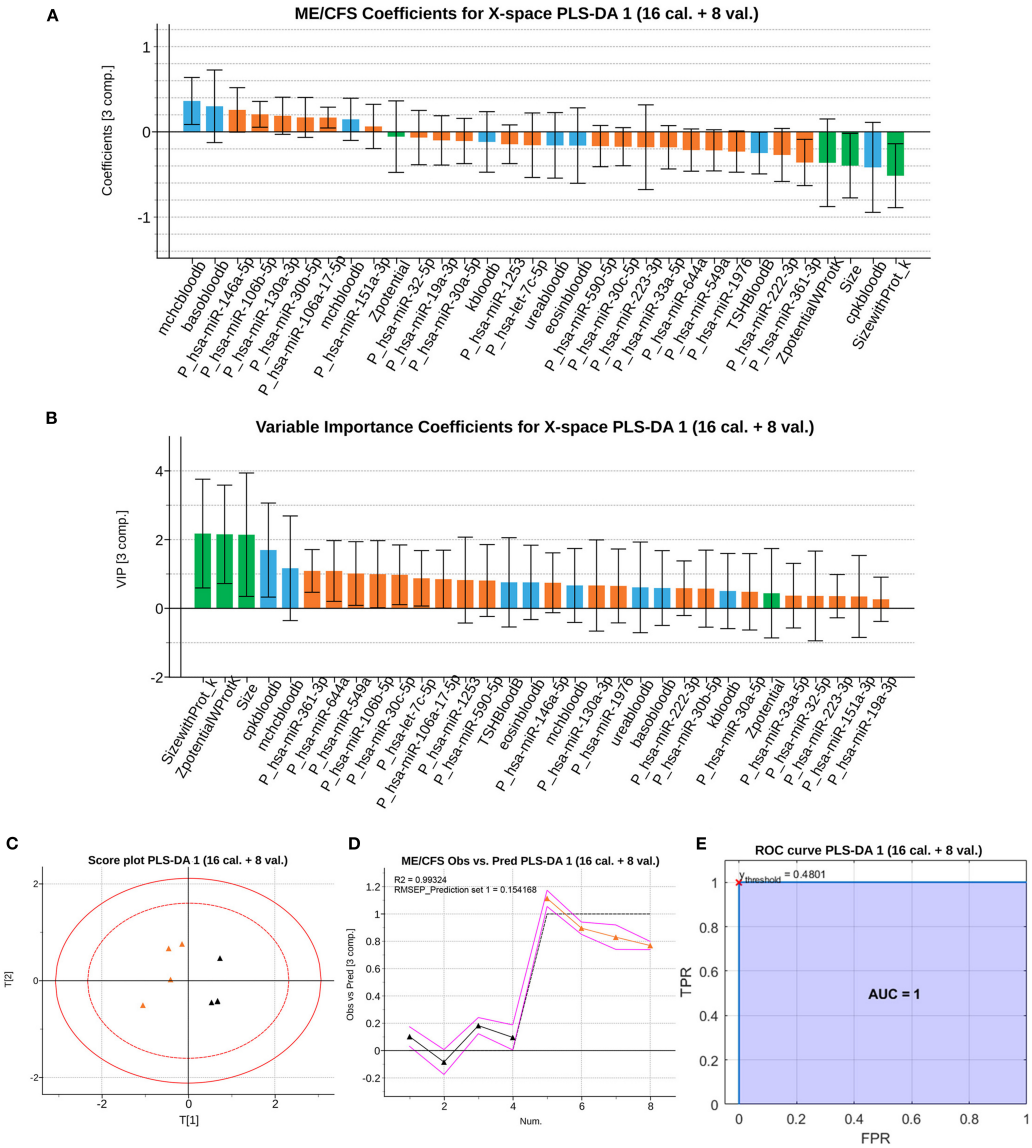
Classification performance of the PLS-DA model with calibration and validation set. **(A)** ME/CFS class *b* jackknife coefficients for the X subspace using the calibration dataset. The color code corresponds the block to which each variable belongs, being those analytical variables (blue), PBMCs' miRNAs (orange) and EVs' characteristics (green). Jackknife confidence intervals were calculated at a 95% confidence level. **(B)** *VIP* coefficients with jackknife confidence intervals at 95% of confidence for the X subspace using the calibration dataset. Data set legends can be consulted on Supplementary Table 1. The color code for each variable block is the same as in the rest of the figures. **(C)** Score plot, of the 1st and 2nd components (horizontal and vertical, respectively) for the validation samples. **(D)** Observed vs. Prediction results for the validation samples shows the class prediction with 95% confidence intervals (magenta lines) using three components. The color code is orange for ME/CFS patients, and black markers for Healthy Controls (HCs). RMSEE stands for Root Mean Square Error of Estimation. **(E)** ROC curve for the classification of the validation observations with the trained dataset. The red cross locates the optimal performance point (maximum specificity and sensitivity) using the classification threshold at 0.4801.

The *b* coefficients obtained are almost of the same order, according to their importance, but with wider confidence intervals (Figures 2A,B). This is caused by the removal of the validation samples from the training set, decreasing the sample size and leading to an increase in model uncertainty. Once the model is fitted, the observations of the validation set are projected onto the latent subspace, obtaining their correspondent scores and predictions (Figures 2C,D). These results support the validity of the model developed in the Section ME/CFS Modeling With PLS-DA for the diagnosis of severe ME/CFS patients. The ROC curve for the validation samples (Figure 2E) shows a perfect discrimination (AUC=1) when the PLS-DA model is used to classify new individuals as healthy or those affected by severe ME/CFS. This means that the model maintains the perfect detection of ME/CFS patients (perfect sensitivity) while keeping the perfect discrimination of healthy controls (specificity = 1).

### Raman Spectroscopy Analysis Supports Composition Differences in ME/CFS Plasma EVs

Intrigued by the fact that four out of the six physical associated parameters of EVs (EV concentration, size, and *z*-potential obtained with or without proteinase K pretreatment), corresponding to the size and zeta potential of vesicles [as described in Almenar-Pérez et al. (12)] were discriminating features selected by our initial PLS-DA model (Figures 1A, 2A), we decided to further explore the differential nature of ME/CFS EVs by Raman spectroscopy analysis, an approach that has proven to differentiate EVs from various cell sources (34) and has been successfully used to detect ME/CFS specific changes in PBMCs (35).

**Figure 3:**
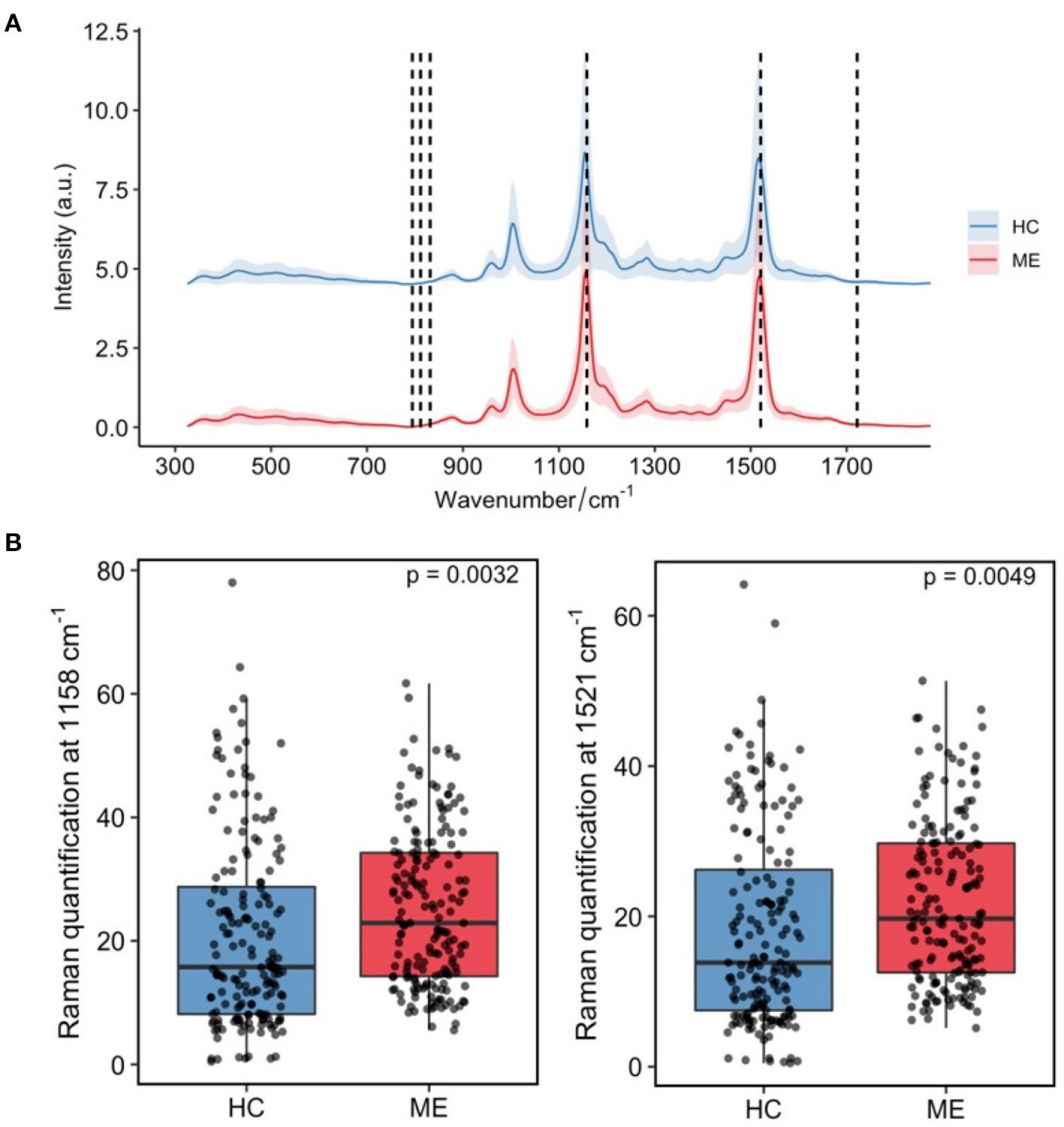
Main differences in plasma derived-EVs Raman spectroscopic profiles from ME/CFS (ME, red, *N* = 15) and matched healthy subjects (HC, blue, *N* = 15). **(A)** Mean profile plot values with indication of chemical nature of peaks with prominent differences. **(B)** Relative quantification of carotenoids by integrating Raman bands at 1,158 cm^−1^ (*p* = 0.0032) (left) and 1,521 cm^−1^ (*p* = 0.0049) (right). The quantification results were represented as box plots and sample mean of the ME/CFS group (ME) compared with the healthy control's (HC) (*N* = 15/group) by using Welch's two sample *t*-test for unequal variance.

Raman analysis of the 15 severe ME/CFS cases and 15 HC EVs isolated from aliquots of the plasma used in our earlier study (12), clearly show prominent Raman bands at 1,158 and 1,521 cm^−1^ (Figure 3A; Supplementary Table 2). These bands are characteristic of carotenoids with the C–C stretching mode (coupled with C–H in-plane bending) contributing to the 1,158-cm^−1^ band and the C = C stretching mode of the conjugated chain in carotenoids contributing to the 1,510-cm^−1^ band (36). Further quantification of results for these two bands are shown in Figure 3B, illustrating a significant higher content of carotenoids in ME/CFS patients than in HCs (*p* = 0.003 and *p* = 0.005).

### ME/CFS Classification Model Based on Raman Spectral Fingerprints

To further investigate the power of Raman spectroscopy to differentiate patients from healthy subjects, we used again PLS-DA as a classifier solely based on the whole Raman spectra. We also compared PLS-DA with a modified version of the LDA, RF, and SVMs to evaluate if there were more suitable techniques to classify individuals using only the Raman spectra as an input.

#### PLS-DA Model

To evaluate the biomarker value of the observed differential Raman peaks we applied PLS-DA analysis to Raman data. Complete spectra of individuals within each group are represented in Supplementary Figure 4 (HCs in blue and ME/CFS patients in red). As it can be appreciated, signals were already preprocessed and can be directly used for their further analysis with multivariate statistics techniques. Due to a slight (though not relevant) mismatch in the wavelengths of different records, abscises axes in Supplementary Figure 4 are representing wavelength bins that contain the signal recorded for wavelengths within each interval.

An PLS-DA model was fitted to determine if the spectra contained information able to discriminate between the groups. The wavelength intervals that carry discriminant information, should appear with significant *b* or *VIP* coefficients. The first PLS-DA model (*R*^2^ of 23.95% and *Q*^2^ of 16.33%) was not able to separate the groups since many variables are non-statistically significant in terms of the *b* and *VIP* coefficients. This can be observed from the high number of jackknife confidence intervals for the VIPs below the *VIP* = 1 threshold (see Supplementary Figure 5A), and by the jackknife confidence intervals for the *b* coefficients that contain a zero value (see Supplementary Figure 5B).

**Figure 4:**
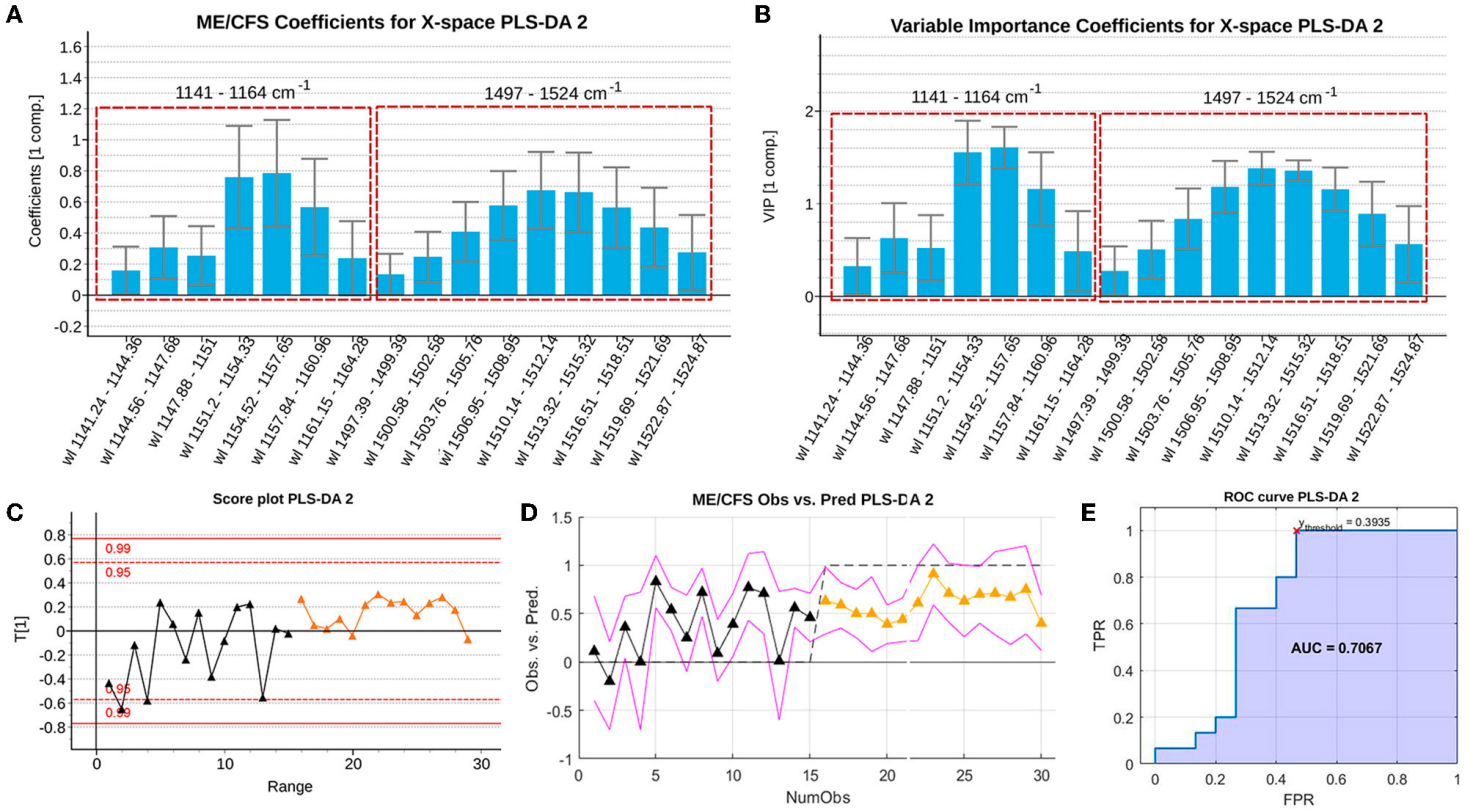
Summary of the *b* PLS coefficients **(A)**, the *VIP* coefficients **(B)**, the scores **(C)**, the observed vs. predicted values (RMSEE stands for Root Mean Square Error of Estimation). **(D)** and ROC curve **(E)** of the depurated PLS-DA model with the Raman spectroscopy data. The red cross locates the optimal performance point (maximum specificity and sensitivity) using the classification threshold at 0.3935. Data set legends can be consulted on Supplementary Table 1. Black triangles represent healthy controls, whereas orange triangles represent ME/CFS cases.

All non-significant variables according to these parameters were deleted and the model re-estimated. The resulting model selects only one latent variable, slightly increasing its goodness of fit (*R*^2^ of 29.57%) and of prediction (*Q*^2^ of 26.36%). The classification performance of the depurated PLS-DA model (Figure 4) is illustrated in the observed vs. predicted values (Figure 4D) and in its corresponding ROC curve generated using the 3-fold cross validation scheme (Figure 4E). The model reaches an optimal AUC value of 0.7067 setting a threshold of 0.3935 on the predicted response. Despite the poor performance of the model in terms of classification, there might still be statistically significant information which could be useful in discriminating the two groups.

Note that Figures 4A,B display the *b* PLS and *VIP* coefficients for the prediction of the ME/CFS class, respectively. Variables with positive *b* coefficients, indicate wavelengths of the spectrum for which the ME/CFS patients show a statistically significant higher signal when compared to the signal of HCs. Relevant variables according to the *b* PLS coefficients highlight the importance of the characteristic peaks on which the previous univariate analysis was focused. In the *b* bar graph, the left window encloses the region close to the 1,158 cm^−1^ peak, while the right window encloses wavelengths close to the 1,521 cm^−1^ peak.

#### Comparison of PLS-DA Model to Other Classification Models

**Figure 5:**
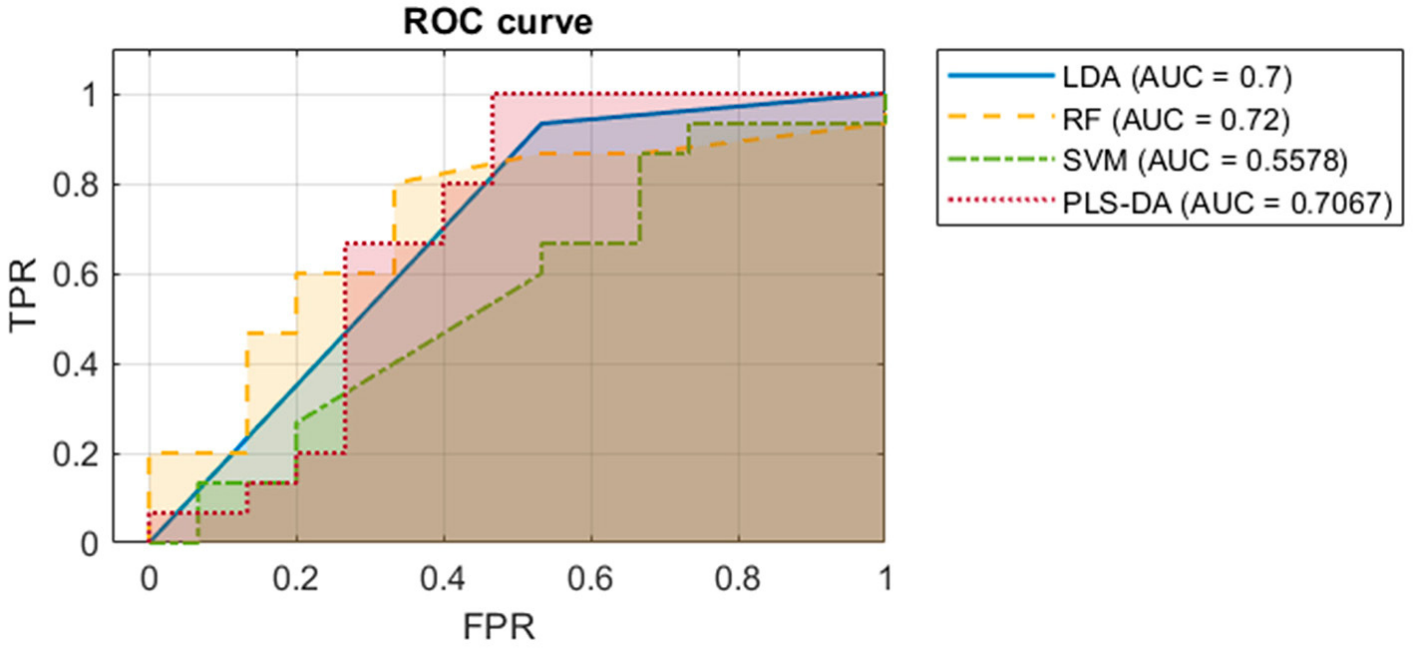
ROC curves with their AUCs of the four models classifying ME/CFS or HC based on their Raman spectra. The ROC curve is plotted with true positive rate against false positive rate.

To further investigate the value of the Raman spectra in differentiating severe ME/CFS patients from HCs, we trained three other binary classification models. We used an adaptation of linear discriminant analysis (LDA) for cases with more variables than observations, a random forest (RF), and a support vector machine (SVM). Some of these techniques (such as RF and SVMs) can model non-linearities which could improve the outcome yielded by the PLS-DA model. The same 3-fold cross validation setup as for the PLS-DA model was used, to make results comparable. All ROC curves with their respective AUCs were obtained, as presented in Figure 5. Further information about the comparison between these models can be found on the Supplementary Methods.

These results suggest that the Raman spectroscopy data by itself does not hold enough information to accurately discriminate between ME/CFS patients and healthy subjects: to achieve a 100% of true positive rate, classifiers would produce a high rate of false positives. However, AUC values close to 0.7 (Figure 5) suggest that EVs might still be representing part of the phenotype of the disease. For this reason, we proposed the last model, combining our initial biomarkers and EV Raman profiles.

### Refinement of the Initial PLS-DA Model With EV Raman Profiles

The results of Raman spectrometry analysis show that to be developed as a more comprehensive diagnostic tool the use of further information is required. Therefore, we proceeded to reanalyze our first multi-block PLS-DA model (Figure 2) to check if the relevant Raman wavelengths selected by the PLS-DA model on the spectroscopy data (Figure 4) could be useful predictors when combined with the previously identified biomarkers.

To study this possibility, we fitted a PLS-DA model using the selected variables from the former PLS-DA model, adding the key differential wavelengths from our PLS-DA analysis of Raman spectroscopy data. It is important to highlight that the adequacy of this approach resides in the fact that the samples used to generate the two models came from the same blood samples. The reason for maintaining the use of PLS-DA, was that according to the previous results, it was a technique yielding one of the best classification performances and the only one enabling the interpretation of the discriminant power of the predictors, establishing a set of statistically significant biomarkers.

**Figure 6:**
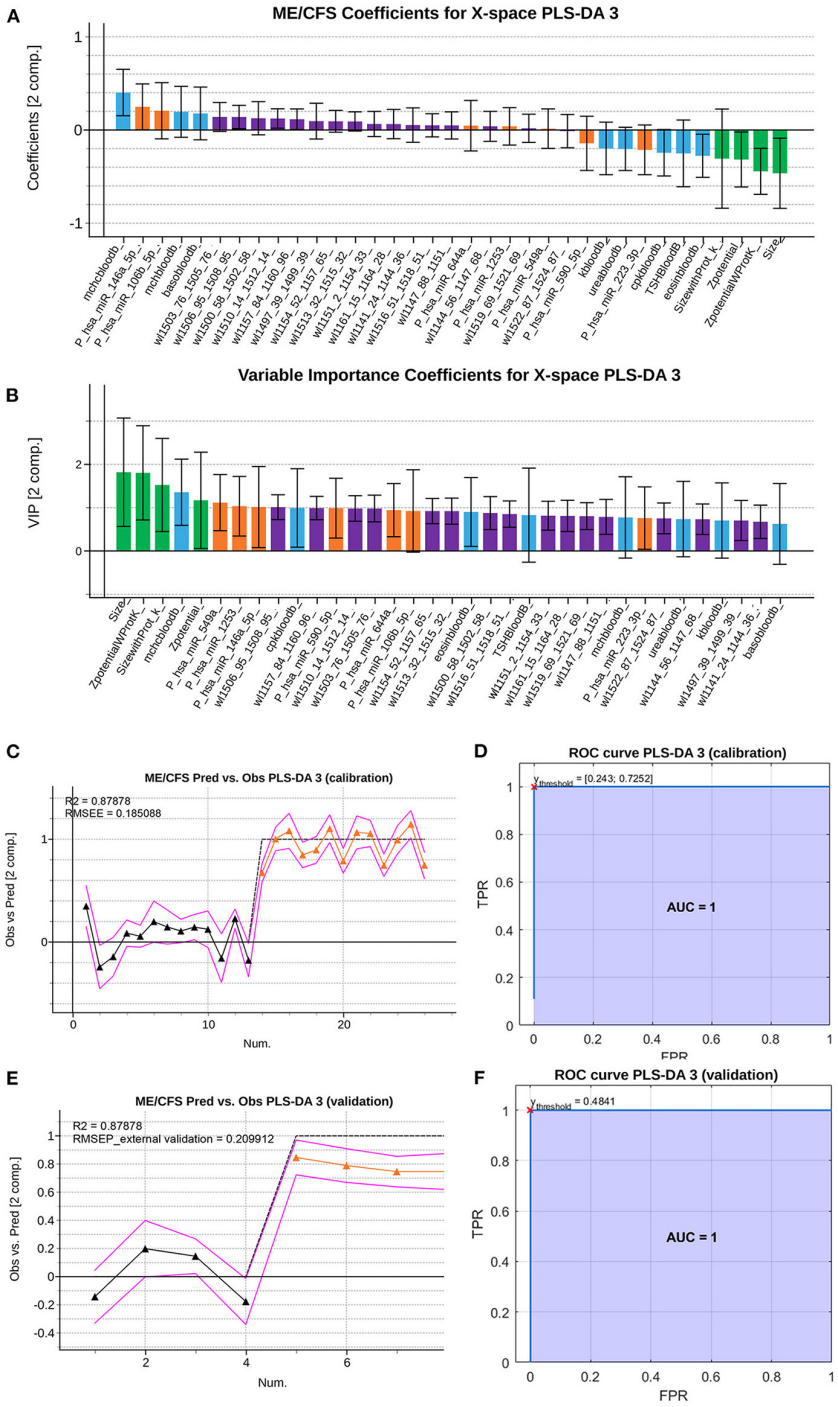
Summary of the *VIP* coefficients **(A)**, *b* PLS coefficients **(B)**, observed vs. predicted values for the training set **(C)**, ROC curve for the training set with red cross indicating the point of optimal performance **(D)** observed vs. predicted values for the validation set (RMSEE stands for Root Mean Square Error of Estimation) **(E)** and ROC curve for the validation set with red cross indicating the point of optimal performance **(F)** of the depurated PLS-DA model with the Raman spectroscopy data. Data set legends can be consulted on Supplementary Table 1. Black triangles represent HCs, whereas orange triangles represent ME/CFS patients. Predictor coefficients in **(A,B)** are colored according to their block of information (blue for analytical features, orange for PBMCs miRs features, green for EVs' features and purple for Raman spectra features).

An initial PLS-DA model was fitted using all observations to allow for the selection of key discriminating variables and removal of potential outliers. The initial fused model selects an optimal number of nine latent variables (*R*^2^ of 99.37% and *Q*^2^ of 81.15%). This model was depurated observation-wise and variable-wise, as previously described. The *b* coefficients and *VIP* coefficients of the final set of selected variables are shown in Figures 6A,B, respectively.

This refined PLS-DA model based on the final set of selected predictors was fitted excluding the observations used for external validation in the first PLS-DA model. The final model obtained presents a similar performance (*R*^2^ of 93.38 and *Q*^2^ of 77.06). Supplementary Figure 6 shows the result of the permutation test performed on the PLS-DA model fitted with the calibration set, proving the statistical significance of the yielded coefficients.

The observed vs. predicted values for the observations in the calibration set (Figure 6C) and in the external validation set, show that classes can be perfectly separated (Figure 6E). This is also illustrated by the ROC curves in Figures 6D,F, showing that a threshold on the predicted outcome of 0.481 yields a perfect classification with an AUC of 1.

Inspecting the *b* PLS and *VIP* coefficients (Figures 6A,B, respectively), although some of the predictors still appear as statistically non-significant, their jackknife confident intervals are almost under or above zero for the *b* coefficients, or almost contain the value *VIP* = 1 for the *VIP* coefficients. This suggests that the width of the confidence intervals might be influenced by the small sample size, which leads to wide jackknife confidence intervals. In conclusion, this final model yields a perfect classification (AUC=1) and has 35 predictors, meaning that some of the most relevant predictors according to the previous PLS-DA model, have been replaced by wavelength intervals of the Raman spectroscopy analysis. Among these relevant wavelengths, both peaks (around 1,158 and 1,521 cm^−1^) hold important information as potential biomarkers. The majority of eliminated predictors from the previous PLS-DA model, carried information about PBMC miRNAs.

**Figure 7:**
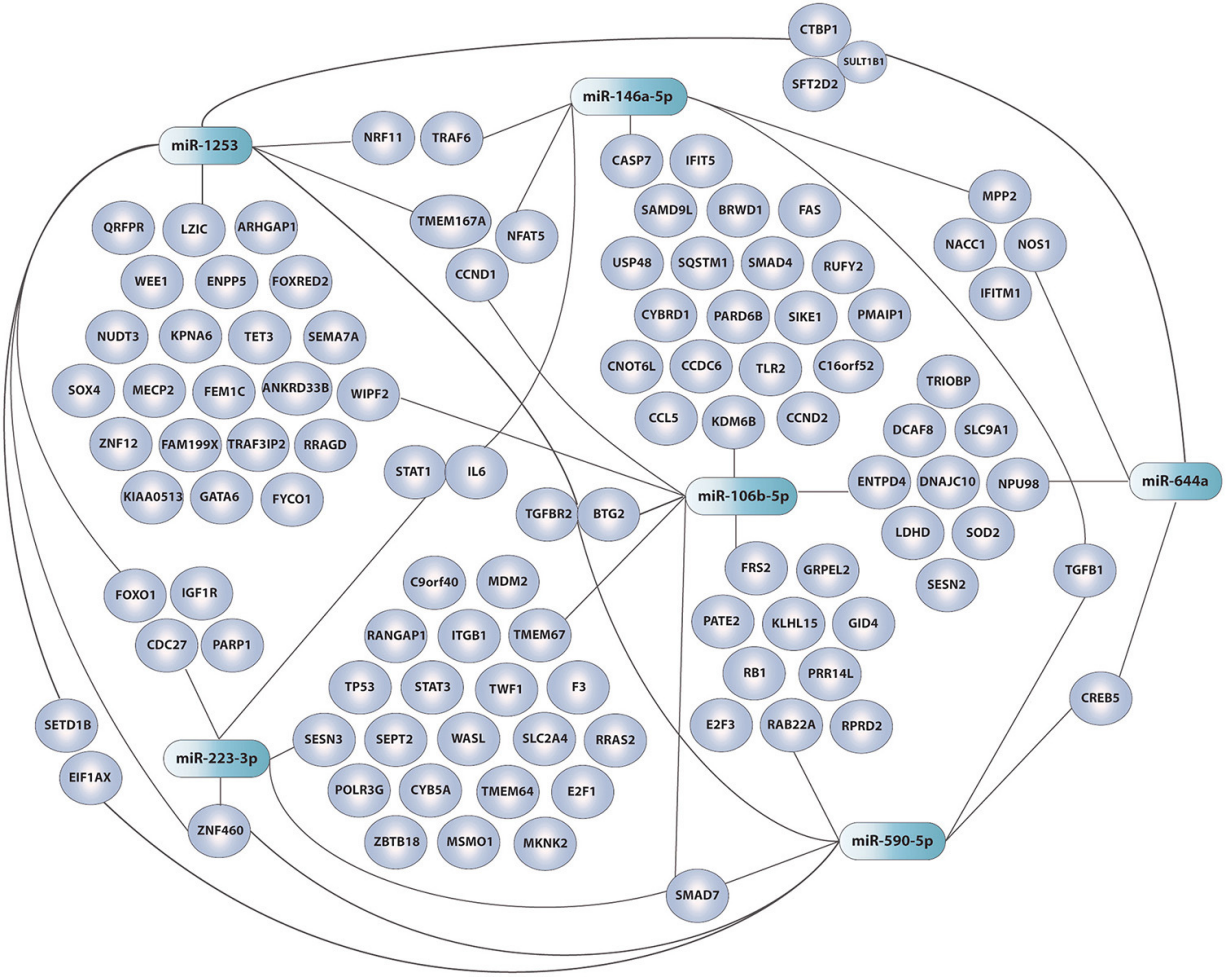
Network of DE miRNAs in ME/CFS PBMCs selected by PLS-DA (shaded green) and their target genes (shaded purple). GO enrichment performed with the miRTargetlink software (33). Adobe Illustrator was the drawing tool used.

GO pathway analysis of DE miRNAs from PBMCs selected by our refined PLS-DA model (Figure 6) show that six out of seven share common gene targets with top cellular functions belonging to immunity, neuroinflammation, and metabolism (Supplementary Table 3; Figure 7), all being widely associated with ME/CFS in the literature.

## Discussion

Because of the lack of an objective diagnostic laboratory test, the diagnosis of ME/CFS is made by ruling out other conditions. ME/CFS patients may not get a diagnosis in many developed countries while in developing countries ME/CFS is still not considered a “real” illness. The burden on patients and their families is enormous.

In 2015 the Institute of Medicine (IOM) in the US (37) informed that ME/CFS is a medical illness and should not be considered a psychiatric condition. In support of IOM conclusions that ME/CFS has a biological basis numerous studies show neurologic (38), immune (39), and metabolic (40) disturbances in these patients. Still, ME/CFS biomarker validation remains an important challenge with many research groups identifying putative diagnostic markers which could help move forward our understanding of the affected pathways in the disease. Research efforts in ME/CFS remain hampered by low numbers of participants in the cohorts studied with disease heterogeneity also playing a role.

The UK National Institute for Health and Care Excellence (NICE) has recently changed the guidelines to treat ME/CFS patients in the NHS (National Health System) (41). The new guidelines do not include graded exercise as a therapeutic strategy. Recent studies have showed that more than 50% of patients either could not start a GET program or failed to complete it, emphasizing the problems of introducing any exercise support program (42, 43). This highlights the urgent need for not only a diagnostic test but the importance of identifying biological/clinical variables able to select patients who are likely to benefit from a particular treatment program. With rising numbers of Long-Covid patients and the possibility that many end-up developing ME/CFS, having good diagnostic test to help patients manage their condition is more important than ever.

Our previous study by Almenar-Pérez et al., although limited in scope by a low number of participants (*N* = 30, 15/group) attempted to improve patient homogeneity by restricting the inclusion of participants to only severe female cases. The selection of severe cases was based on the premise that severity concurs with highest differential biomarker levels. Although the scope of the findings may be limited to this patients' group, it remains possible that the mechanisms and, therefore, the detected biomarkers turn up valid to diagnose moderately or mildly affected patients. A design including the study of a large number of variables encompassing PBMC and EV miRNomes, together with complete blood analytics, thorough patient phenotyping by validated questionnaires, and the study of EV physical features (12) led to the identification of biological differences with limited diagnostic potential at the individual level.

In the current study we combine these variables and add Raman spectroscopic profiling as a new marker of EV function in the same blood samples. By applying PLS-DA analysis to this large set of data: 34 blood analytic variables, 775 different miRNAs being expressed above threshold levels (136 in PBMCs and 639 in EVs), EV concentration, size and z-potential, we identified 32 variables that can effectively differentiate ME/CFS cases from HCs (AUC=1, i.e., sensitivity and specificity = 1) (Figure 1). Moreover, a second model using calibration and validation sets further confirms the effective diagnostic power of the selected variables (Figure 2), with an AUC still equal to 1 (i.e., sensitivity and specificity are perfect). Strikingly, EV physical features, including EV size and z-potential measures were detected by this model as relevant features for the effective diagnosis of patients indicating a potential important role of EVs in ME/CFS.

Although we and others have consistently found higher counts of EVs in different cohorts of ME/CFS patients (12, 44, 45), even by applying different isolation procedures, EV count in the PLS-DA model was not among the 32 features selected that could discriminate severe ME/CFS patients from healthy subjects. The reasons behind this result are not understood at present. However, the fact that increased EV numbers have been reported for other diseases with an inflammatory component (46, 47) may argue for a restricted disease specificity of this feature.

It is worth mentioning that among the blood analytic group of variables the iterative PLS-DA modeling process selected, blood creatine phosphokinase (CK, labeled as cpkbloodb, please see Supplementary Table 1 key tab for variable nomenclature used) level was a feature retrieved with and without the inclusion of Raman data (Figures 1, 2, 6). CK levels being a clinical feature that had been previously reported as a potential biomarker of ME/CFS for showing significant reduced levels in an expanded cohort of patients (48). Highly expressed in muscle, heart, and brain the CK enzyme holds a key role in ATP homeostasis. The low levels found by Nacul et al., possibly reflecting energy dysregulation in these tissues, may be linked to the profound fatigue found in ME/CFS patients with the severe having the lowest levels.

The increased absolute zeta potential values of ME/CFS EVs detected in a previous study by our group (12) suggested differences in the relative abundance of charged groups in their membranes. Modifications of EVs membrane potential has been related to other pathological conditions, including cancer where the change in EV net charge was attributed to a disbalance in the relative abundance of sialic acid (49). Interestingly polysialylation of exosomal membranes has been shown to provide a thermo-protecting effect being able to modulate exosome-plasma membrane interactions and thus their signaling capacity (50). Further evaluation of these modifications present in ME/CFS EVs will be an important component of future studies aimed at determining their functional impacts as proposed in our recent publication (51).

Raman spectroscopy has shown its utility in detecting composition differences in patient's EVs (52, 53) and could be developed as a cost-effective diagnostic method by its ability to identify complex patterns in biological materials. Encouraged by the discriminating potential of this method to unveil composition differences in biological materials, EVs isolated from severe ME/CFS patients which had shown reduced diameter and reduced zeta potential (increased electronegativity) (12), were compared to HC EVs by Raman micro-spectroscopic analysis. The main difference in the EVs Raman spectra between severe ME/CFS patients and HCs related to two carotenoid peaks (Figure 3; Supplementary Figure 4). Zhang et al., have recently found a shift of a peak at 1,553 cm^−1^ (tryptophan/amide II) to 1,528 cm^−1^ (carotenoid) in trophoblast-derived EVs during late stages of pregnancy (54), time at which circulating EVs counts increase and inflammatory responses vary (55, 56).

In the 1970's Raman spectroscopy was used to study the protein properties of red blood cells (RBC) ghosts (57). RBC ghosts are pale cells which turn up on blood smears, coming from the hemolysis of RBCs, are typically linked to disease. Verma and Wallach identified two Raman peaks in RBC ghosts which were later identified as carotenoids (58, 59). Recent studies have showed RBC deformability was reduced in ME/CFS (60). Thus, it is tempting to speculate that the EV differences we are observing by Raman are due to EVs of RBC origin being generated when the RBC are stressed in the patient's circulation. In support of this hypothesis, it is interesting to observe that increased mean corpuscular hemoglobin (mch) and mean corpuscular hemoglobin concentration (mchc), which have been related to decreased deformability of RBCs (61), were identified by our PLS-DA analysis as variables with high discriminant diagnosis capacity (Figure 6; Supplementary Table 1). It seems relevant to mention that Fiedor et al., have recently shown that increased beta-carotene concentration in RBC membranes affect cell's shape, sensitivity to osmolysis and alters hemoglobin-oxygen affinity with potential physiologic implications (62).

Regardless of EV composition differences we were interested in exploring if the Raman spectroscopic data was sufficient to efficiently distinguish ME/CFS cases from HCs. Despite its potential discriminatory capacity of ME/CFS body fluid components (Figures 4, 5), in good agreement with the disease “plasma factor” hypothesis reported by Ron Davis' group at Standford University (63), which is also supported by differences in proteins or lipid plasma levels (64, 65), the diagnostic value of Raman data seems limited when compared to our PLS-DA model including analytic variables, PBMC miRNA profiles and EV features (Figures 1, 2).

It needs to be considered that a particular isolation method used to purify EVs from plasma may lead to the purification of EV sets that may differ from another procedure. Despite the high purity attributed to EVs prepared by ultracentrifugation, this procedure is laborious, and requires both a large volume of fluid and the provision of expensive equipment. A diagnostic method based on EVs requires a much simpler method preferably allowing the analysis of small volumes of fluids without compromising performance. Total Exosome Isolation Reagent (TEIR) was selected from the available kits because according to Helwa et al., it provides higher yields using smaller amounts of plasma when compared to other commercial alternatives or with respect to ultracentrifugation, ultrafiltration, or gel chromatography (66). Moreover, exploratory EV studies using highly purified EV sets (i.e., exosomes) could turn into missing relevant EV subsets, and thus a less restrictive method was preferred.

Unexpectedly our PLS-DA iterative method did not select any of the 639 miRNAs detected above threshold levels in ME/CFS EVs. All miRNAs in our panel of discriminatory measures came from the PBMC's group. Although this may associate with the complexity of ME/CFS, and thus the requirement of features from different compartments for its definition, the possibility that a more selective EV isolation method may render homogenous EV subpopulations with distinctive ME/CFS miRNA profiles cannot be ruled out at present. In support of the first argument, we find that GO pathway analysis of six out of the seven DE miRNAs from PBMCs selected by our PLS-DA model (Figure 6) share common gene targets with top cellular functions belonging to immunity, neuroinflammation, and metabolism (Supplementary Table 3; Figure 7), all being widely associated with ME/CFS in the literature.

In summary, this work describes for the first time an ME/CFS model based on PLS-DA of 32 analytical variables capable of diagnosing the disease with perfect sensitivity and specificity (AUC=1), further confirming the biologic nature of this disease and highlighting the relevance of patient EV features for their diagnosis. An ME/CFS EV Raman spectroscopic fingerprint is also provided, pioneering the potential use of this method for the diagnosis of ME/CFS and for detecting potential RBC defects in severe ME/CFS. Finally, we show that although the diagnostic potential of Raman is limited its simplicity and low amount of sample requirement highlights its potential utility as an early screening tool prior to more comprehensive testing with miRNA's from PBMC's. Moreover, the inclusion of Raman data for the refinement of our previous model, although incapable of increasing the already perfect separation of cases from HCs (AUC=1) (Figures 1, 6), allowed for a significant reduction in the number of PBMC miRNAs from 21 in our initial PLS-DA model (Figures 1, 2) to only 7 in the PLS-DA Raman refined model (Figure 6) (Supplementary Table 1).

The findings obtained in this study are expected to pave the way for unraveling the subjacent disease mechanisms in which EVs and PBMC miRNAs participate with clear implications for the future diagnosis and treatment of ME/CFS, perhaps embracing other patient groups suffering with chronic fatigue.

## Data Availability Statement

The datasets presented in this study are available from the NCBI GEO (Accession number GSE141770) or as Supplementary Material.

## Ethics Statement

The studies involving human participants were reviewed and approved by Public Health Research Ethics Committee DGSP-CSISP, Valencia (Spain), study number UCV_201701 and the UCL Biobank Ethical Review Committee-Royal Free London NHS Foundation Trust (B-ERC-RF), study number EC2017.01. The patients/participants provided their written informed consent to participate in this study.

## Author Contributions

AG-C and AF: data curation, formal analysis, methodology, figure drawing, writing—original draft, review, and editing. EA-P, TY, WH, SH, TL, and LN: formal analysis, investigation, methodology, data curation, and manuscript review. JX: formal analysis, investigation, data curation, methodology, and writing—original draft. KG-O: formal analysis, investigation, data curation, figure drawing, and manuscript review. KM: formal analysis, investigation, data curation, writing—original draft, and manuscript review. EO: conceptualization, main funding acquisition, supervision, formal analysis, investigation, data curation, writing—original draft, and manuscript review. All authors contributed to the article and approved the submitted version.

## Funding

This study was funded by the Generalitat Valenciana AICO grant number 2020/254 and by a Ramsay Fund MEA (ME Association, UK) grant to EO; by the Research and Development Support Program of the Universitat Politècnica de València (PAID-01-17) to AF; by the Star Exclusivas SL grant to the UCV Gene expression and immunity group. Erasmus staff mobility to KM and EO. SH was supported by a UK Spine Bridge support grant and KG-O by the Generalitat Valenciana ACIF2021/179 grant. Funders were not involved in any of the research stages.

## Conflict of Interest

The authors declare that the research was conducted in the absence of any commercial or financial relationships that could be construed as a potential conflict of interest.

## Publisher's Note

All claims expressed in this article are solely those of the authors and do not necessarily represent those of their affiliated organizations, or those of the publisher, the editors and the reviewers. Any product that may be evaluated in this article, or claim that may be made by its manufacturer, is not guaranteed or endorsed by the publisher.
