# Supplementary material for: Diagnosis of Myalgic Encephalomyelitis/Chronic Fatigue Syndrome With Partial Least Squares Discriminant Analysis: Relevance of Blood Extracellular Vesicles
Source: Front Med (Lausanne). 2022 Apr 1;9:842991. doi: 10.3389/fmed.2022.842991 (PMC9011062; doi:10.3389/fmed.2022.842991)
Supplement: Supplementary file 1 [file Data_Sheet_1.PDF]

Supplementary Material

1 Supplementary Methods

Supplementary note on training and validation of binary classifiers for discriminant analysis using Raman spectra.

In this supplementary note we address some further details about the methodology followed to compare the four binary classifiers: PLS-DA, LDA, RF and SVM.

ROC curves for all four methods were obtained following a 3-fold cross validation approach. This way, all observations had a prediction obtained without using them for the model fitting. This let us preserve 2/3 of the data for the training, leaving the other 1/3 of observations for the external validation. Some classifiers offer to a certain extent the interpretation of the set of discriminant predictors. However, this was not feasible for all of them, as we address in this supplementary note. The following points detail some technical aspects considered for each classifier:

- **Partial Least Squares – Discriminant Analysis.** The PLS-DA model was the one previously depurated (k = 16 variables). The depuration was performed by iteratively eliminating predictors with statistically unsignificant VIP and b PLS coefficients, as explained in the main text of this work.
- **Adaptation of Linear Discriminant Analysis to deal with more variables than observations.** The LDA model was fitted using all the predictors (k = 1018 variables). One mathematical aspect of the LDA, is that it needs to invert the covariance matrix of the predictors. This step is compromised when the number of variables is higher than the number of observations, as in this case (1018 variables >> 30 observations). Nonetheless, there are numerical solutions implemented to enable the obtention of a solution. However, this can come at the cost of losing coherence in the coefficients of the model. In fact, as for the PLS-DA, a previous depuration step based on the coefficients of the discriminant function, was considered. However, as it can be seen in the following figure, there was too much numerical instability to perform this depuration. This can be appreciated by the high number of coefficients containing the zero value, which means that depending on the fitting round, these coefficients could be either positive or negative. The error bars indicate the range of variation of the coefficient, i.e., its minimum and maximum along the three cross validation folds.

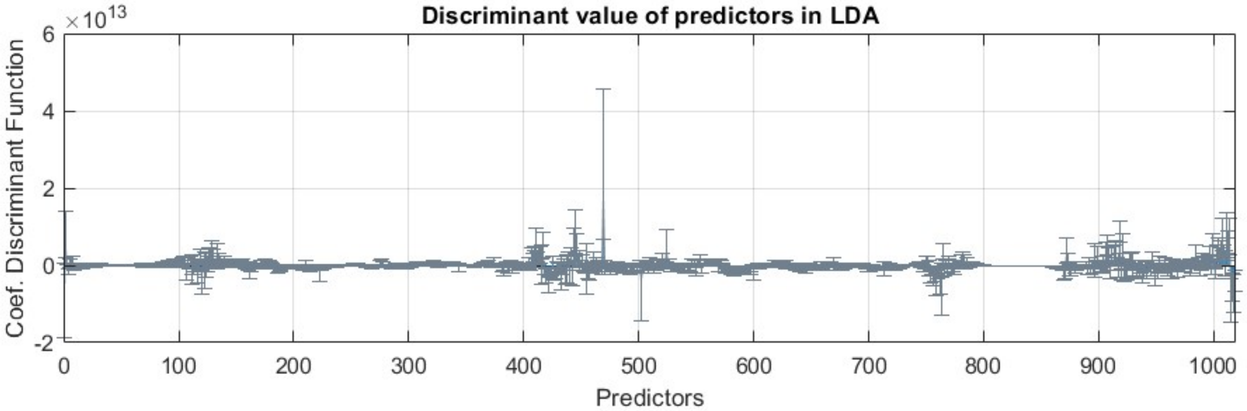

Figure 1. Coefficients of the predictors in the discriminat functions fitted for each fold of the cross-validation scheme used for the Raman spectra classifiers.

- **Random Forest.** The RF model was fitted using all the predictors (k = 1018 variables). The hyperparameters optimization was performed by the MATLAB classification learner app. This optimization consists of finding the model configuration that minimizes the cross-validated misclassification rate. In this case the Variable Importance metrics could be used as well to refine the model or to know which variables hold more discriminant power. In this case, variable importance is measured by a permutation test. This test randomly shuffles the values of a given variable and measures the difference in the classification error due to the fact of altering that variable. Figure 2 illustrates the variable importance for predictors whose importance metrics were above zero over the three cross-validation folds. The fact that they present a positive average importance means that their permutation causes a detriment on the model performance.

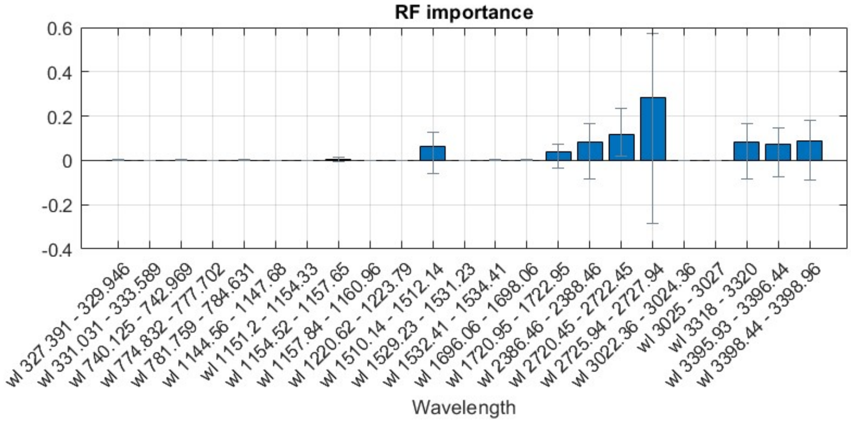

Figure 2. Variable importance metrics obtained for the Random Forest model based on the Raman spectra.

As it can be seen, there are some variables selected that indicate the importance of wavelengths close to the 1158 cm^{-1} peak and to the 1521 cm^{-1} peak. However, not all the important variables agree with the ones selected by the PLS-DA model, and the meaning of their relevance according to the RF model, is beyond further study and understanding. However, the fact that their minimum and maximum

values (limits of the error bars) have different signs, suggests a lack of coherence in the predictors' importance and that their relevance might not be statistically significant.

- **Support Vector Machine.** SVM model was fitted using all the hyperparameters' optimization of the MATLAB classification learner app. This optimization consists of finding the model configuration that minimizes the cross-validated misclassification rate. The optimizer selected a "linear" kernel function for the first fold, a "gaussian" kernel function for the second fold and a "polynomial" kernel function for the third fold. This incoherence on the optimal SVM, suggests a lack of information within the Raman spectra to build a stable and reliable classifier, which is aligned with the conclusions obtained with the rest of classifiers.

Supplementary univariate tests

The following table presents the results of the univariate t-student tests performed for each biomarker of the final PLS-DA model, with predictors from the analytics (Block A), the PBMCs' miR (Block B), the EVs' characteristics (Block C) and the Raman spectra (Block D).

Table 1. Results of the univariate t-tests on the biomarkers of the final PLS-DA model.

Block	Variable	m(sd) all	m(sd) HC	m(sd) Pat	t-student (Pat - HC)	p-value	C.I. (Pat - HC)
A	mchbloodb	29,42 (1,52)	28,91 (1,7)	29,93 (1,17)	1,7866	0,0866	[-0,15882; 2,205]
	mchcbloodb	326,31 (11,4)	320,69 (12,07)	331,92 (7,54)	2,8446	**<0,01**	[3,08222; 19,3793]
	eosinbloodb	0,24 (0,2)	0,29 (0,22)	0,19 (0,18)	-1,3041	0,2046	[-0,26422; 0,059603]
	basobloodb	0,04 (0,02)	0,03 (0,02)	0,04 (0,02)	1,2684	0,2168	[-0,0057888; 0,02425]
	kbloodb	4,13 (0,47)	4,17 (0,49)	4,1 (0,46)	-0,3698	0,7148	[-0,45562; 0,31716]
	ureabloodb	4,11 (1,09)	4,44 (0,83)	3,78 (1,25)	-1,5761	0,1281	[-1,5101; 0,20236]
	cpkbloodb	74 (27,22)	85,85 (23,43)	62,15 (26,27)	-2,4267	**<0,05**	[-43,8422; -3,54242]
	TSHBloodB	1,69 (0,77)	1,9 (1,01)	1,48 (0,33)	-1,4278	0,1662	[-1,0309; 0,18779]
B	P_hsa-miR-1253	153,31 (75,65)	179,31 (94,17)	131,31 (49,34)	-1,6004	0,1238	[-110,1875; 14,19851]
	P_hsa-miR-106b-5p	205,44 (61,16)	179,55 (42,4)	227,34 (67,37)	2,0326	0,0543	[-0,969631; 96,5331]
	P_hsa-miR-549a	155,42 (63,74)	180,86 (80,49)	133,89 (35,95)	-1,8974	0,0710	[-98,294; 4,36768]
	P_hsa-miR-590-5p	135,83 (37,25)	152,72 (44,46)	121,54 (23,01)	-2,2084	**<0,05**	[-60,4491; -1,89925]
	P_hsa-miR-146-5p	649,97 (168,19)	573,64 (105,5)	714,56 (187,3)	2,2115	**<0,05**	[8,768621; 273,0796]
	P_hsa-miR-223-3p	20963,66 (6860,08)	21512,46 (8491,02)	20499,29 (5441,4)	-0,3536	0,7270	[-6955,7044; 4929,3513]
	P_hsa-miR-644a	177,92 (70,14)	199,12 (68,96)	159,98 (68,6)	-1,3893	0,1786	[-97,5651; 19,2845]
C	Zpotential	-11,04 (1,71)	-10,45 (1,83)	-11,62 (1,43)	-1,8299	0,0797	[-2,5038; 0,15048]
	ZpotentialWProtK	-9,76 (1,44)	-8,95 (1,01)	-10,56 (1,38)	-3,3929	**<0,01**	[-2,5832; -0,62912]

	Size	98,19 (13,39)	105,23 (10,19)	90,37 (12,49)	-2,8550	**<0,05**	[-25,8472; -3,87943]
	SizewithProt_k	95,13 (7,25)	98,5 (7,82)	91,39 (4,41)	-2,4013	**<0,05**	[-13,359; -0,863183]
	wl1141.24 - 1144.36	0,23 (0,04)	0,21 (0,04)	0,24 (0,04)	2,1889	**<0,05**	[0,0018923; 0,064369]
	wl1144.56 - 1147.68	0,29 (0,06)	0,26 (0,06)	0,31 (0,05)	2,3145	**<0,05**	[0,0055327; 0,096652]
	wl1147.88 - 1151	0,31 (0,06)	0,28 (0,07)	0,34 (0,03)	2,5803	**<0,05**	[0,010657; 0,09585]
	wl1151.2 - 1154.33	0,4 (0,12)	0,34 (0,13)	0,46 (0,07)	2,7853	**<0,05**	[0,029338; 0,1972]
	wl1154.52 - 1157.65	0,39 (0,12)	0,32 (0,12)	0,45 (0,07)	3,4429	**<0,01**	[0,05232; 0,20893]
	wl1157.84 - 1160.96	0,32 (0,09)	0,26 (0,08)	0,37 (0,05)	3,9559	**<0,01**	[0,052323; 0,16648]
D	wl1161.15 - 1164.28	0,23 (0,05)	0,21 (0,06)	0,26 (0,04)	2,7582	**<0,05**	[0,013303; 0,09239]
	wl1497.39 - 1499.39	0,17 (0,03)	0,16 (0,03)	0,19 (0,02)	2,4845	**<0,05**	[0,0045316; 0,049007]
	wl1500.58 - 1502.58	0,22 (0,04)	0,2 (0,04)	0,24 (0,03)	3,3668	**<0,01**	[0,018164; 0,075713]
	wl1503.76 - 1505.76	0,27 (0,06)	0,24 (0,06)	0,31 (0,03)	4,0213	**<0,01**	[0,036035; 0,11203]
	wl1506.95 - 1508.95	0,32 (0,08)	0,27 (0,07)	0,37 (0,04)	4,2150	**<0,01**	[0,050489; 0,14737]
	wl1510.14 - 1512.14	0,35 (0,09)	0,3 (0,09)	0,41 (0,05)	3,9438	**<0,01**	[0,054073; 0,1728]

In the violin plots, the blue violin plot (ClassNum = 0) is for Healthy Control individuals, whereas the red violin plot (ClassNum = 1) is for ME/CFS Patients. Only statistically significant biomarkers according to the t-student tests (Spplementary Table 1) are displayed. On one hand, biomarkers with higher levels for the blue violin plot, are intended to be protection factors, i.e., the higher their value, the lower the risk of having ME/CFS. On the other hand, biomarkers with higher red violin plots, are risk factors, i.e., the higher their value, the higher the risk of having ME/CFS.

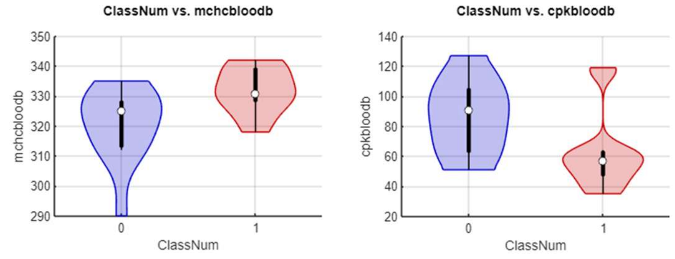

Figure 3. Violin plot for mchcbloodc and cpkblood.

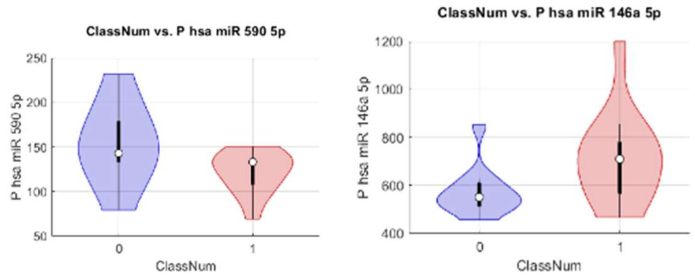

Figure 4. Violin plot for P_hsa_miR-590-5p and P_hsa-miR-146a-5p.

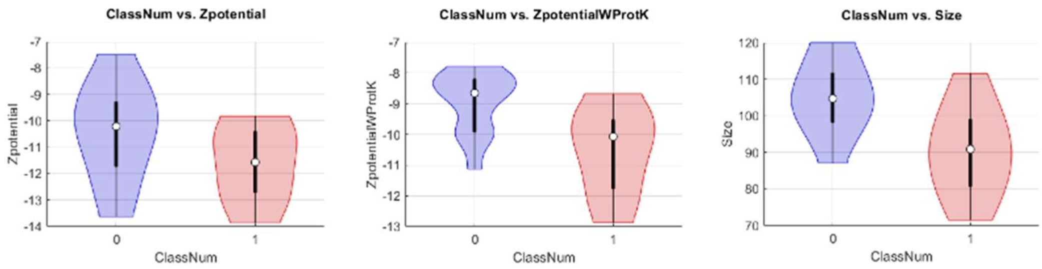

Figure 5. Violin plots for Zpotential, ZpotentialwProtK and Size.

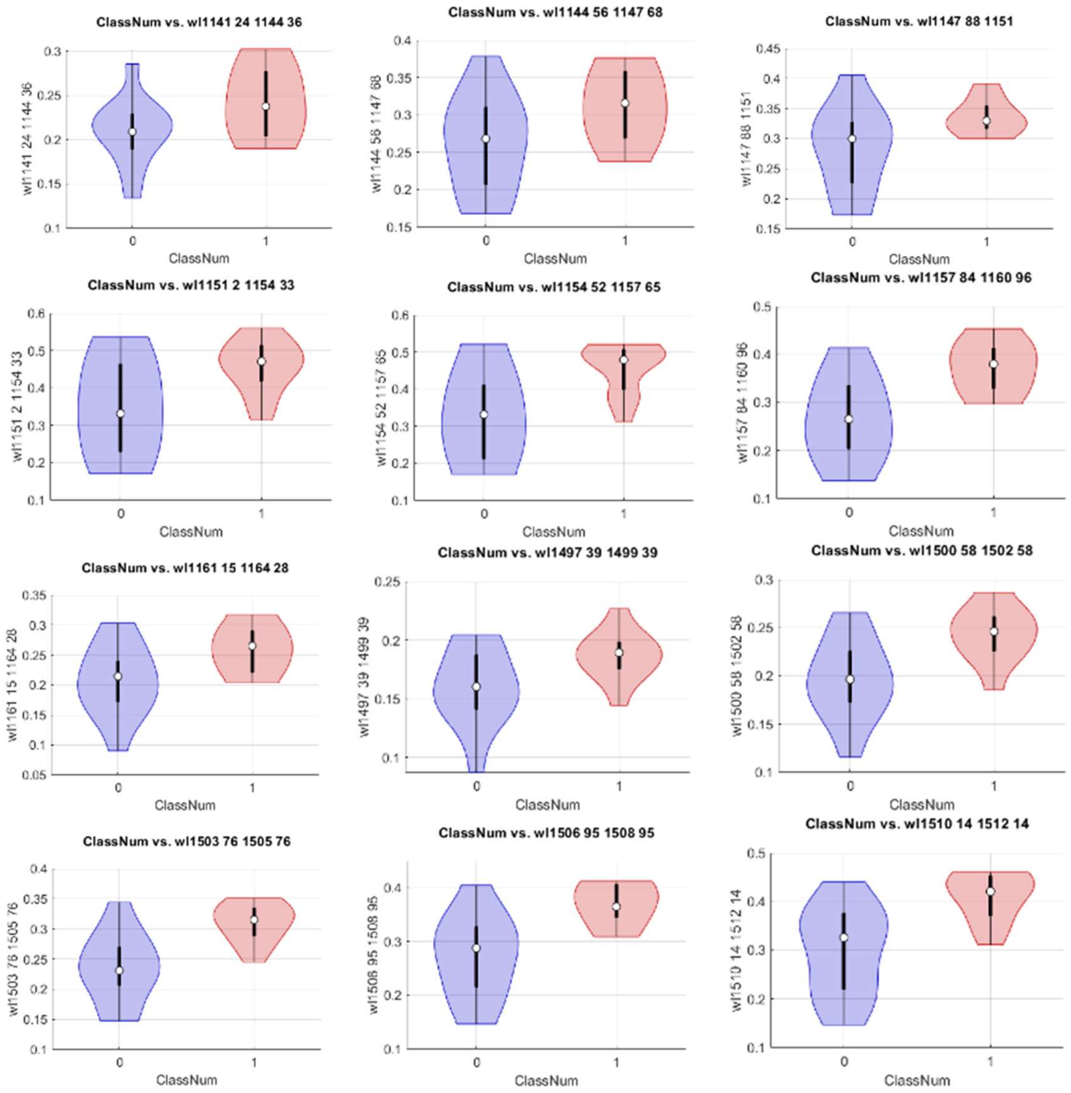

Figure 6. Violin plots for the statistically significant wavelengths of the Raman

2 Supplementary Figures

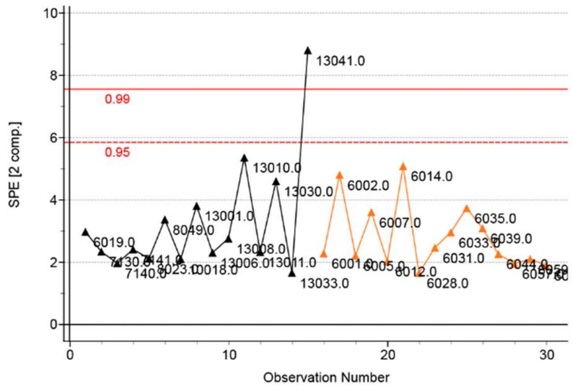

Supplementary Figure S1. Squared Prediction Error (SPE) for the observations (i.e., patients) with the initial PLS (Partial Least Squares)-DA (Differential Analysis) multiblock model. Black triangles are healthy controls, whereas orange triangles are ME/CFS patients. The observation with ID 13041 is an example of outlier overpassing the SPE control limit (red lines).

A

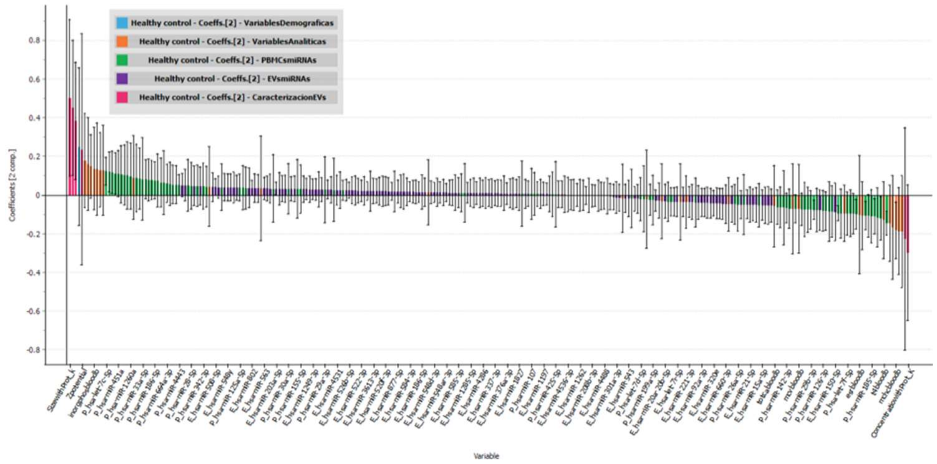

B

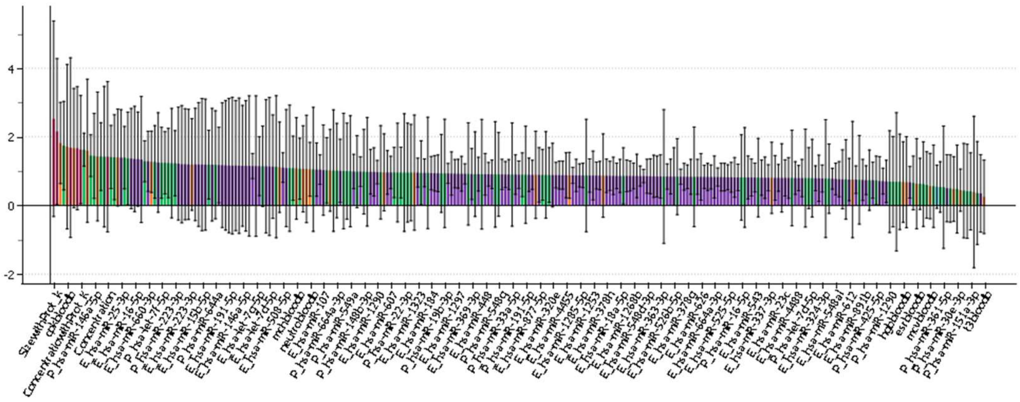

Supplementary Figure S2. (A) Set of jackknife b coefficients for each variable and the class "Healthy Control" with its 95% jackknife confidence interval, obtained with the full data set. Each variable block is represented by one color (demographic variables in blue, analytic variables in orange, PBMCs' miRNAs variables in green, EVs' miRNAs variables in purple and EVs' characterization in pink). **(B)** VIP coefficients of the predictor variables for the PLS-DA model with all the set of predictors. The colour code is the same as in the b coefficients figure.

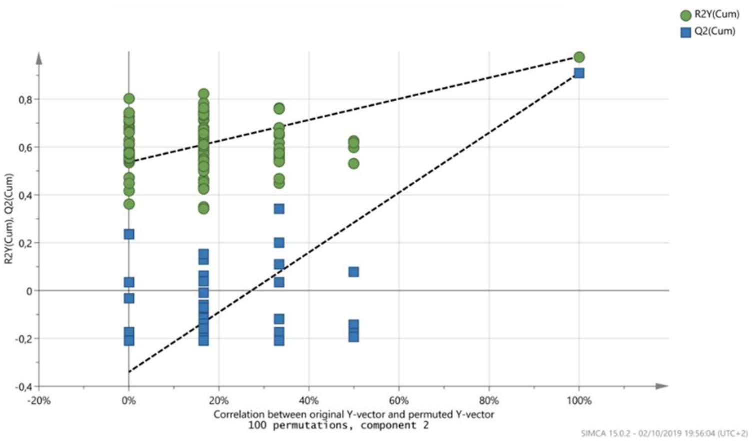

Supplementary Figure S3. Permutation test for the variable filtered PLS-DA model. The values of the model coefficients are expressed in the vertical axis, whereas in abscises it is expressed the correlation between the real response vector and the different permutated versions.

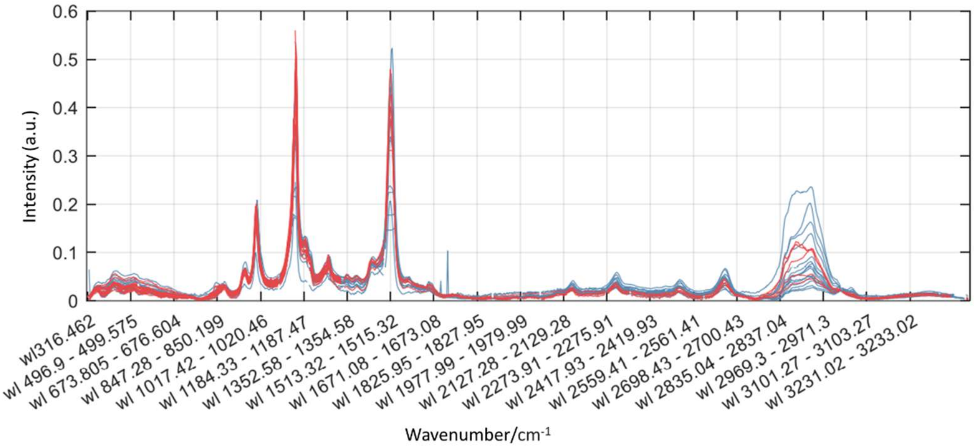

Supplementary Figure S4. Complete Raman spectra of EVs isolated from plasma from 15 ME/CFS patients (red) and 15 matched control individuals (blue).

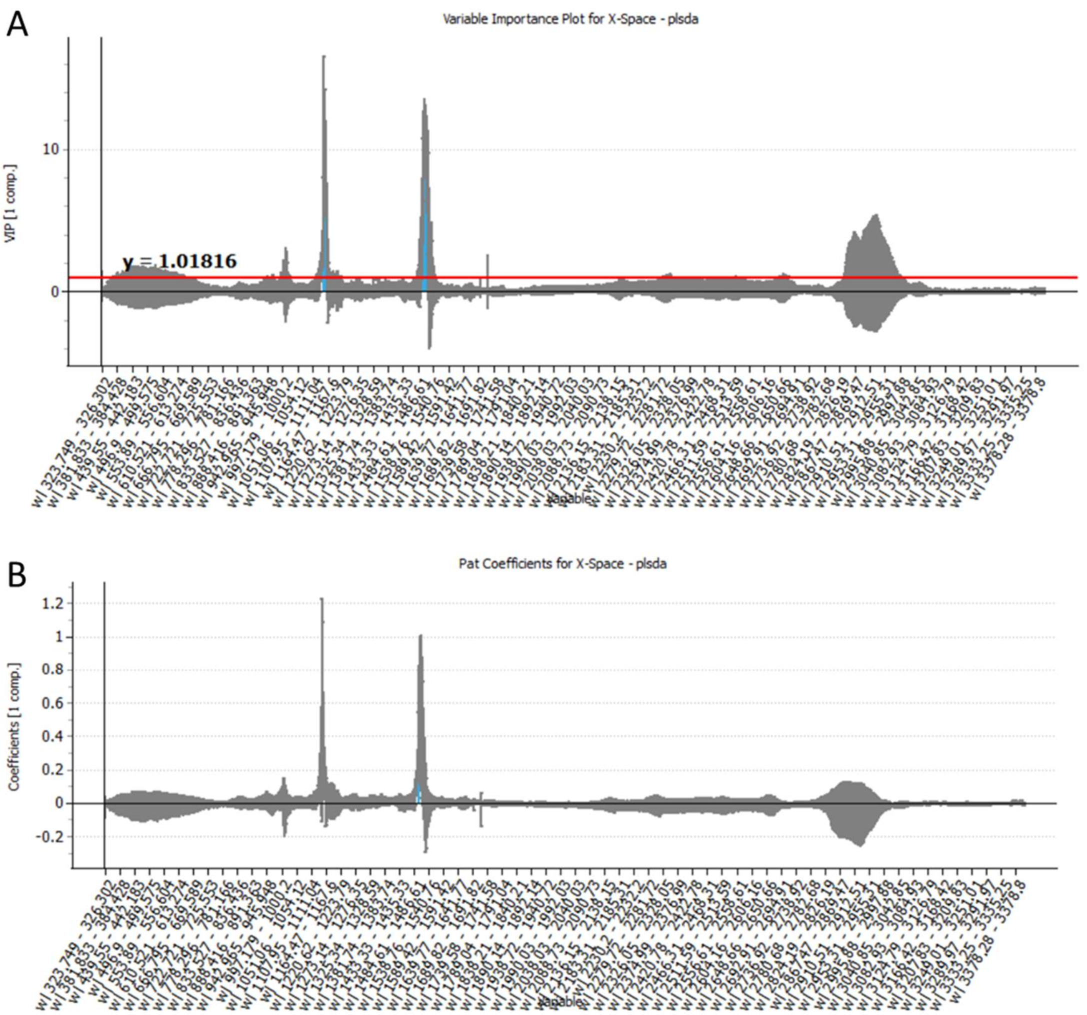

Supplementary Figure S5. (A) VIP coefficients for the predictors (wavelength intervals) of the PLS-DA model. **(B)** B coefficients for the predictors (wavelength intervals) of the PLS-DA model.

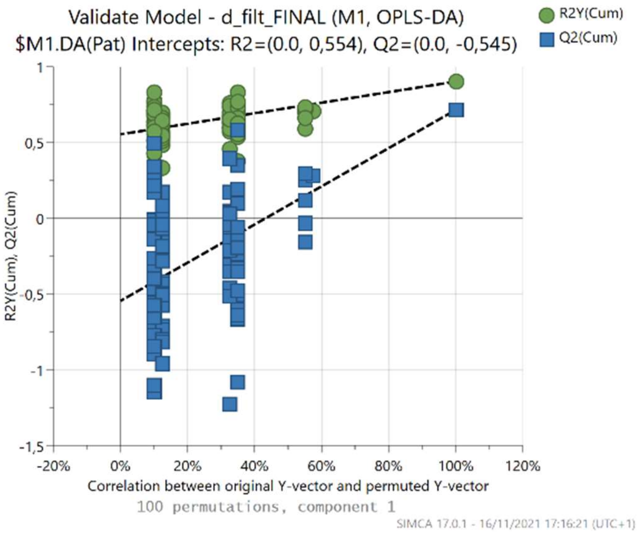

Supplementary Figure S6. Permutation test for the depurated PLS-DA model based on the fused database. The values of the model coefficients are expressed in the vertical axis, whereas in abscises it is expressed the correlation between the real response vector and the different permutated versions.
