# Supplementary material for: Diagnosis of Myalgic Encephalomyelitis/Chronic Fatigue Syndrome With Partial Least Squares Discriminant Analysis: Relevance of Blood Extracellular Vesicles
Source: Front Med (Lausanne). 2022 Apr 1;9:842991. doi: 10.3389/fmed.2022.842991 (PMC9011062; doi:10.3389/fmed.2022.842991)
Supplement: Supplementary file 4 [file Table_3.pdf]

Supplementary Table S3. Fifty top GO categories by gene targets of at least 2 DE discriminant miRNAs from PBMCs

GO Subcategory	P-adjusted	Q-value	miRNAs/precursors
chemokine receptor binding	1,59E-03	1,59E-03	hsa-miR-223-3p, hsa-miR-146a-5p
cytokine receptor binding	1,59E-03	1,59E-03	hsa-miR-590-5p, hsa-miR-223-3p, hsa-miR-146a-5p
CCR5 chemokine receptor binding	2,00E-03	2,00E-03	hsa-miR-223-3p, hsa-miR-146a-5p
detection of biotic stimulus	2,60E-03	2,60E-03	hsa-miR-223-3p, hsa-miR-146a-5p
interferon-alpha production	2,60E-03	2,60E-03	hsa-miR-223-3p, hsa-miR-146a-5p
positive regulation of T-helper 2 cell cytokine production	2,60E-03	2,60E-03	hsa-miR-223-3p, hsa-miR-146a-5p
positive regulation of T cell mediated immunity	2,60E-03	2,60E-03	hsa-miR-223-3p, hsa-miR-146a-5p
positive regulation of interferon-alpha production	2,60E-03	2,60E-03	hsa-miR-223-3p, hsa-miR-146a-5p
positive regulation of neuroinflammatory response	2,60E-03	2,60E-03	hsa-miR-223-3p, hsa-miR-146a-5p
positive regulation of type 2 immune response	2,60E-03	2,60E-03	hsa-miR-223-3p, hsa-miR-146a-5p
signaling receptor activator activity	3,97E-03	3,97E-03	hsa-miR-223-3p, hsa-miR-146a-5p
transforming growth factor beta receptor cytoplasmic mediator activity	3,97E-03	3,97E-03	hsa-miR-106b-5p, hsa-miR-590-5p, hsa-miR-146a-5p
T-helper 2 cell cytokine production	5,18E-03	5,18E-03	hsa-miR-223-3p, hsa-miR-146a-5p
adaptive immune response	5,18E-03	5,18E-03	hsa-miR-223-3p, hsa-miR-146a-5p
extracellular matrix assembly	5,18E-03	5,18E-03	hsa-miR-590-5p, hsa-miR-146a-5p
microglial cell activation	5,18E-03	5,18E-03	hsa-miR-223-3p, hsa-miR-146a-5p
monocyte chemotaxis	5,18E-03	5,18E-03	hsa-miR-223-3p, hsa-miR-146a-5p
myeloid dendritic cell differentiation	5,18E-03	5,18E-03	hsa-miR-590-5p, hsa-miR-146a-5p
neural precursor cell proliferation	5,18E-03	5,18E-03	hsa-miR-590-5p, hsa-miR-223-3p, hsa-miR-146a-5p
positive regulation of adaptive immune response	5,18E-03	5,18E-03	hsa-miR-223-3p, hsa-miR-146a-5p
positive regulation of myeloid cell differentiation	5,18E-03	5,18E-03	hsa-miR-590-5p, hsa-miR-223-3p, hsa-miR-146a-5p
positive regulation of nitric-oxide synthase biosynthetic process	5,18E-03	5,18E-03	hsa-miR-223-3p, hsa-miR-146a-5p
regulation of macrophage activation	5,18E-03	5,18E-03	hsa-miR-223-3p, hsa-miR-146a-5p
regulation of neural precursor cell proliferation	5,18E-03	5,18E-03	hsa-miR-590-5p, hsa-miR-223-3p, hsa-miR-146a-5p
regulation of vascular endothelial growth factor production	5,18E-03	5,18E-03	hsa-miR-223-3p, hsa-miR-146a-5p
vascular endothelial growth factor production	5,18E-03	5,18E-03	hsa-miR-223-3p, hsa-miR-146a-5p
Rho protein signal transduction	5,84E-03	5,84E-03	hsa-miR-223-3p, hsa-miR-146a-5p
adaptive immune response based on somatic recombination of immune receptors built from immunoglobulin superfamily domains	5,84E-03	5,84E-03	hsa-miR-223-3p, hsa-miR-146a-5p
inflammatory cell apoptotic process	5,84E-03	5,84E-03	hsa-miR-223-3p, hsa-miR-146a-5p
macrophage activation	5,84E-03	5,84E-03	hsa-miR-223-3p, hsa-miR-146a-5p
neuroinflammatory response	5,84E-03	5,84E-03	hsa-miR-223-3p, hsa-miR-146a-5p
positive regulation of macrophage differentiation	5,84E-03	5,84E-03	hsa-miR-590-5p, hsa-miR-146a-5p
positive regulation of vascular endothelial growth factor production	5,84E-03	5,84E-03	hsa-miR-223-3p, hsa-miR-146a-5p
regulation of T cell cytokine production	5,84E-03	5,84E-03	hsa-miR-223-3p, hsa-miR-146a-5p
regulation of adaptive immune response	5,84E-03	5,84E-03	hsa-miR-223-3p, hsa-miR-146a-5p
regulation of neuroinflammatory response	5,84E-03	5,84E-03	hsa-miR-223-3p, hsa-miR-146a-5p
signaling receptor activity	6,93E-03	6,93E-03	hsa-miR-223-3p, hsa-miR-146a-5p
cardiac chamber development	7,08E-03	7,08E-03	hsa-miR-590-5p, hsa-miR-223-3p, hsa-miR-146a-5p
regulation of reactive oxygen species metabolic process	7,08E-03	7,08E-03	hsa-miR-590-5p, hsa-miR-223-3p, hsa-miR-146a-5p
positive regulation of T cell cytokine production	7,33E-03	7,33E-03	hsa-miR-223-3p, hsa-miR-146a-5p
positive regulation of glial cell differentiation	7,33E-03	7,33E-03	hsa-miR-223-3p, hsa-miR-146a-5p
regulation of extracellular matrix assembly	7,33E-03	7,33E-03	hsa-miR-590-5p, hsa-miR-146a-5p
response to immobilization stress	7,33E-03	7,33E-03	hsa-miR-590-5p, hsa-miR-223-3p

Supplementary Table S3. Fifty top GO categories by gene targets of at least 2 DE discriminant miRNAs from PBMCs

cytokine activity	7,90E-03	7,90E-03	hsa-miR-223-3p, hsa-miR-146a-5p
T cell cytokine production	9,25E-03	9,25E-03	hsa-miR-223-3p, hsa-miR-146a-5p
regulation of bone resorption	9,25E-03	9,25E-03	hsa-miR-223-3p, hsa-miR-146a-5p
regulation of glial cell differentiation	9,25E-03	9,25E-03	hsa-miR-223-3p, hsa-miR-146a-5p
response to xenobiotic stimulus	9,25E-03	9,25E-03	hsa-miR-223-3p, hsa-miR-146a-5p
defense response to virus	1,20E-02	1,20E-02	hsa-miR-106b-5p, hsa-miR-146a-5p
heart valve development	1,33E-02	1,33E-02	hsa-miR-590-5p, hsa-miR-223-3p, hsa-miR-146a-5p
regulation of carbohydrate metabolic process by regulation of transcription from RNA polymerase II promoter	1,44E-02	1,44E-02	hsa-miR-106b-5p, hsa-miR-223-3p
